# A temporal beta‐diversity index to identify sites that have changed in exceptional ways in space–time surveys

**DOI:** 10.1002/ece3.4984

**Published:** 2019-02-18

**Authors:** Pierre Legendre

**Affiliations:** ^1^ Département de sciences biologiques Université de Montréal Montréal Québec Canada

**Keywords:** B–C plots, beta diversity, space–time analysis, statistical power, temporal beta diversity, temporal beta diversity index, type I error

## Abstract

**Aim:**

This paper presents the statistical bases for temporal beta‐diversity analysis, a method to study changes in community composition through time from repeated surveys at several sites. Surveys of that type are presently done by ecologists around the world. A temporal beta‐diversity Index (TBI) is computed for each site, measuring the change in species composition between the first (T1) and second surveys (T2). TBI indices can be decomposed into losses and gains; they can also be tested for significance, allowing one to identify the sites that have changed in composition in exceptional ways. This method will be of value to identify exceptional sites in space–time surveys carried out to study anthropogenic impacts, including climate change.

**Innovation:**

The null hypothesis of the TBI test is that a species assemblage is not exceptionally different between T1 and T2, compared to assemblages that could have been observed at this site at T1 and T2 under conditions corresponding to H_0_. Tests of significance of coefficients in a dissimilarity matrix are usually not possible because the values in the matrix are interrelated. Here, however, the dissimilarity between T1 and T2 for a site is computed with different data from the dissimilarities used for the T1–T2 comparison at other sites. It is thus possible to compute a valid test of significance in that case. In addition, the paper shows how TBI dissimilarities can be decomposed into loss and gain components (of species, or abundances‐per‐species) and how a B–C plot can be produced from these components, which informs users about the processes of biodiversity losses and gains through time in space–time survey data.

**Main conclusion:**

Three applications of the method to different ecological communities are presented. This method is applicable worldwide to all types of communities, marine, and terrestrial. R software is available implementing the method.

## 1 INTRODUCTION

Community ecology is the scientific study of the interactions among species in natural communities, their distribution through space and their evolution through time, and of the relationships between the species and their environment. Changes in community composition through time are at the center of community ecology research (McEwan, Dyer, & Pederson, 2011; Pickett, Collins, & Armesto, 1987; Vellend, 2016), including the nature of these changes (e.g., gains and losses of species) and their quantitative importance.

In the spatial context, the variation in community composition among sites in a region of interest has been called beta diversity by Whittaker (1972), who defined the well‐known concepts of alpha, beta, and gamma diversities. In recent years, interest of ecologists and managers has turned to the study of the temporal variation in community composition, either at a single site or at a series of sites repeatedly surveyed across time. This temporal variation was called *temporal beta diversity* by Legendre and Gauthier (2014) and Shimadzu, Dornelas, and Magurran (2015). Temporal variation can be the result of gradual or abrupt changes in environmental conditions, including man‐induced alterations such as the present worldwide climate warming.

Statistical inference methods have been proposed for the analysis of temporal changes in community composition. For example, (a) the temporal convergence or divergence in composition of a set of communities can be studied by testing for differences in multivariate dispersion among surveys (Anderson, 2006); (b) shifts in mean composition of monitored communities can be statistically tested using multivariate analysis of variance procedures (Anderson, 2001; Legendre & Anderson, 1999), including null models (Schaefer, Gido, & Smith, 2005); (c) the interaction between the factors space and time and other complex spatio‐temporal structures can be studied and tested for significance (Angeler, Viedma, & Moreno, 2009; Legendre, Cáceres, & Borcard, 2010; Legendre & Gauthier, 2014).

In several application fields, researchers want to compare observations made at several sites and at two different times. The question of interest is as follows: are there sites where the difference between survey times seems exceptionally large? These sites would be worth examining in more detail to identify and compare the causes of the differences. Here are some examples. (a) In paleoecology, comparison of ancient and modern diatom communities preserved in lake sediment cores may indicate areas where acute anthropogenic processes have singularly changed the surrounding land use (e.g., Winegardner, Legendre, Beisner, & Gregory‐Eaves, 2017). (b) When a strong natural or man‐made or environmental impact has taken place at a known point in time and an ecological community had been surveyed ahead of the impact, ecologists may survey that community again to determine how it was affected by the impact, and then how it may have recovered in later surveys (e.g., Legendre & Salvat, 2015). (c) In community ecology, when studying a permanent stem‐mapped forest dynamics plot divided into quadrats, examining surveys made at two different times may indicate sections of the forest that have been exceptionally affected by a disturbance, for example, a climatic or anthropogenic event (e.g., Legendre & Condit, 2019).

This paper describes a method to test, for several sampling units (objects), the differences between data vectors corresponding to observations made at time 1 (abbreviated T1) and time 2 (T2). I will refer to these objects as sites in this paper, although they may be of other natures, for example, experimental enclosures. A dissimilarity *D* computed between times T1 and T2 for a site, using community composition (occurrences, frequencies or biomass) or gene frequency data, is called a *temporal beta‐diversity Index* (TBI); it measures the change in community composition (or temporal beta diversity) from T1 to T2. A change through time is directional; species presences, species abundances, or gene frequencies may have been gained and/or lost between T1 and T2. So, it will be of interest to examine the loss and gain components of the TBI indices, in addition to the TBI index values and their significance.

**Figure 1:**
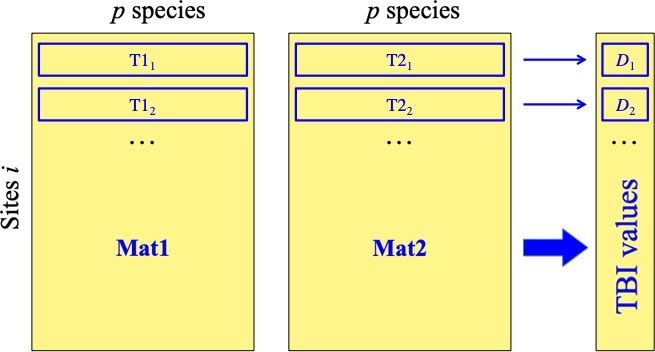
Schematic representation of the first step of the method. Data in matrices **Mat.1** (for Time 1) and **Mat.2** (for Time 2) are used to compute a vector of TBI dissimilarities *D_i_* for all sites *i* (data rows). For example, for site *i* = 1, vectors T1_1_ and T2_1_ are compared to compute *D*_1_, the dissimilarity between data at time 1 (T1) and time 2 (T2)

The observed data, assembled in matrices **Mat.1** for time T1 and **Mat.2** for T2 (Figure 1), may be of different kinds; in landscape ecology and genetics, the data are community composition or population gene frequencies observed at different sites, the same in the two surveys. (a) The null hypothesis (H_0_) to be tested in the statistical testing part of the method is that a site is not exceptionally different in community composition between T1 and T2, for presence–absence or abundance data, compared to assemblages that could have been observed at this site at T1 and T2 under conditions corresponding to H_0_. An exceptionally different site is a site with an index of dissimilarity between T1 and T2 that has an extreme value in the upper tail of the distribution of TBI index values. This value may also have been produced by a different (e.g., ecological) process than the one having generated most other values in the distribution. To determine how extreme a value has to be in the reference distribution before it is considered extreme, we will rely on the usual significance levels (e.g., 5%, 1%, etc.) of statistical analysis. (b) In the data representation part of the method, we will use the species losses and gains between T1 and T2 (for presence–absence or abundance data) to uncover the main ecological changes that have taken place between T1 and T2 in the study area or in subsets (geographic or environmental) of that area.

The paper is organized into sections as follows: Methods (Section 2), Numerical simulations (Section 3), TBI analysis of environmental or trait data (Section 4), Ecological applications (Section 5) where the method is illustrated by three case studies, and Concluding remarks and prospective applications (Section 6).

## 2 METHODS

The proposed method consists basically in the following analyses of temporal variation, detailed in the following subsections. (2.1) Computation and testing: (2.1.1) a temporal beta‐diversity Index (TBI*_i_ = D_i_*) is computed for each site *i* between the data vectors corresponding to T1 and T2 (Figure 1), using an appropriate dissimilarity index (*D*). Then (2.1.2), when it is pertinent to the problem at hand, the indices can be tested for significance using a permutational procedure. (2.2) *Partitioning dissimilarities into losses and gains*: four of the *D* indices that can be used in this type of analysis (two indices for abundance and the corresponding two indices for presence–absence data) also allow the computation of species losses and gains at each site between T1 and T2. These statistical elements provide users with detailed information, at the site level, about the response of the community to the event or change that occurred between T1 and T2. (2.3) The loss and gain statistics can be used together to draw *B–C* *plots*, which illustrate whether the temporal changes at the various sites are dominated by gains or by losses. (2.4) The *Software* subsection lists the R software available to carry out the analyses.

### 2.1 Temporal beta‐diversity Indices: computation and testing

Since Whittaker (1972), ecological dissimilarities have been used to measure beta diversity among sampling units. Koleff, Gaston, and Lennon (2003) reviewed 24 beta‐diversity indices proposed in the literature while Legendre and De Cáceres (2013) described 14 properties of 11 dissimilarity indices that are appropriate for beta‐diversity studies. The Ružička dissimilarity (1958) was identified by Podani, Ricotta, and Schmera (2013) and by Legendre (2014) as another appropriate index for beta‐diversity studies.

Some of these indices are used as TBI indices in this paper: the percentage difference (Odum, 1950; sometimes abbreviated to %diff in the present section of the paper; this index is also known as the Bray‐Curtis dissimilarity; see Legendre & De Cáceres, 2013, Table 1, about the application of the anteriority rule for the name of this index) and Ružička (1958) indices, as well as corresponding forms for presence–absence data, known as the Sørensen and Jaccard indices. Indices in this group of four contain in their equations the quantities *B* and *C*, which represent the species loss and gain components of these dissimilarities. The chord, Hellinger, and log‐chord distances, which are widely used by community ecologists, are also applicable as TBI indices, although they cannot be decomposed into losses and gains. All these indices can be subjected to the test procedure described in Section 2.1.2.

**Table 1:** The dissimilarities (top panel) and *p*‐values (lower panel) associated with the tests of significance of the distances between T1 (survey #4) and T2 (survey #11), for 12 mesocosms (M1 to M12) shown in order of increased insecticide doses

Mesocosms Treatment, μg/L	M1 0	M2 0	M3 0	M4 0	M5 0.1	M6 0.1	M7 0.9	M8 0.9	M9 6	M10 6	M11 44	M12 44
*TBI dissimilarity values*												
%difference *D*	0.433	0.449	0.405	0.459	0.496	0.439	0.488	0.485	0.474	0.621	**0.735**	**0.672**
Ružička *D*	0.604	0.620	0.576	0.630	0.663	0.610	0.656	0.653	0.643	0.766	**0.847**	**0.804**
Chord *D*	0.806	0.836	0.688	0.774	0.872	0.777	0.884	0.895	0.807	0.981	**1.144**	**1.075**
Hellinger *D*	0.859	0.863	0.807	0.860	0.922	0.848	0.930	0.895	0.887	1.072	**1.194**	**1.101**
Log.chord *D*	0.862	0.865	0.814	0.865	0.929	0.854	0.933	0.899	0.899	1.078	**1.200**	**1.106**
Euclidean *D*	26.33	26.55	22.63	22.44	27.24	24.36	26.43	27.12	23.27	24.82	29.79	27.72
*p‐Values corrected for multiple testing*												
%difference *D*	1.000	1.000	1.000	1.000	1.000	1.000	1.000	1.000	1.000	0.192	0.001*	0.020*
Ružička *D*	1.000	1.000	1.000	1.000	1.000	1.000	1.000	1.000	1.000	0.182	0.001*	0.019*
Chord *D*	1.000	1.000	1.000	1.000	1.000	1.000	1.000	1.000	1.000	0.675	0.001*	0.023*
Hellinger *D*	1.000	1.000	1.000	1.000	1.000	1.000	1.000	1.000	1.000	0.119	0.001*	0.032*
Log.chord *D*	1.000	1.000	1.000	1.000	1.000	1.000	1.000	1.000	1.000	0.119	0.001*	0.038*
Euclidean *D*	1.000	1.000	1.000	1.000	1.000	1.000	1.000	1.000	1.000	1.000	0.290	1.000

Note

The *p*‐values (9,999 random permutations) were corrected for multiple testing (Holm correction). The significant *p*‐values at the 0.05 level (marked with an asterisk) and the corresponding TBI indices are in bold. The maximum possible value is 1 for the %difference and Ružička dissimilarities, and $\sqrt{2}$ = 1.4142 for the chord, Hellinger, and log.chord distances. The Euclidean distance does not have an upper bound.

Tests of significance for dissimilarity coefficients (*D*) are usually not possible because the *D* values in a dissimilarity matrix are obtained from the computation of an index between all pairs of objects, for example, sites in ecology (their number is *n*), and are thus interrelated, each site contributing to (*n*–1) of the dissimilarities in the half‐matrix of dissimilarities. In the T1–T2 comparisons for individual sites, however, the dissimilarity between T1 and T2 for a site is computed for the data from that site only, and these differ from the data involved in the T1–T2 comparisons of other sites. Hence, it would be possible to compute a valid test of significance in that case (Section 2.1.2).

#### 2.1.1 Computation of temporal beta‐diversity Indices

Consider two data matrices, **Mat.1** and **Mat.2**, about the same objects observed at times T1 and T2; each matrix has *n* sites (with indices *i*) in rows and the same *p* variables (e.g., species, with indices *j*) in columns (Figure 1). Individual values may be noted *y_ij_*.1 for T1 and *y_ij_*.2 for T2. Compute the dissimilarity *D*(**y** *_i_*.1, **y** *_i_*.2) between the row vectors of values, **y** *_i_*.1 and **y** *_i_*.2, for each site *i*. These dissimilarities form a vector of length *n*, which is the number of sites.

The percentage difference dissimilarity (*D*_%diff_; method “%difference” in the R function TBI.R, also known as the Bray‐Curtis index in other computer packages), and the Ružička dissimilarity (*D*_Ruz_; method “ruzicka” in the R function; see *Software* in Section 2.4 below) can be used for beta‐diversity assessment. They are obtained by computing a dissimilarity function (equations shown below). With presence–absence data, the percentage difference produces the (1 – *S*_Sørensen_) dissimilarity whereas the Ružička dissimilarity produces (1 – *S*_Jaccard_), where *S* designates a similarity index.

The chord, Hellinger, and log‐chord distances are members of the Box–Cox family of distances (Legendre & Borcard, 2018). They are classical indices for beta‐diversity studies (Legendre & De Cáceres, 2013). Their calculation involves two steps: first the calculation of a transformation of each data row (i.e., the chord transformation, the Hellinger transformation, or the transformation log(*y* + 1) followed by the chord transformation; Legendre & Borcard, 2018), followed by calculation of the Euclidean distance. These indices, as well as the Euclidean distance itself, are also implemented in the TBI.R function and will be used in the simulations and in Ecological application 1 below, although they are less interesting for the comparison of community composition matrices than the percentage difference and Ružička indices, which provide additional information about losses and gains of species. The indices in the Box–Cox family are available in the computer function for TBI analysis to ensure compatibility with other multivariate analyses that users may want to do using these popular indices.

When the percentage difference or the Ružička dissimilarity is used as TBI indices, one can compute two derived indices to study the directionality of the change through time at each site, as proposed by Legendre and Salvat (2015). Consider data vectors **y**_1_ and **y**_2_ corresponding to the multi‐species observations at T1 and T2 for a focal site of interest. The following calculations can be done on these vectors:

- *A_j_* is the part of the abundance of species *j* that is common to the two survey vectors: *A_j_* = min(*y*_1_ *_j_*, *y*_2_ *_j_*). *A* is the sum of the *A_j _*values for all species. It represents the unscaled *similarity* between two surveys.
- *B_j_* is the part of the abundance of species *j* that is higher in survey 1 than in survey 2: *B_j_* = (*y*_1_ *_j_* – *y*_2_ *_j_*) if *y*_1_ *_j_* > *y*_2_ *_j_*; *B_j_* = 0 otherwise. *B* is the sum of the *B_j _*values for all species. It is the unscaled sum of *species losses* between T1 and T2.
- *C_j_* is the part of the abundance of species *j* that is higher in survey 2 than in survey 1: *C_j_* = (*y*_2_ *_j_* – *y*_1_ *_j_*) if *y*_2_ *_j_* > *y*_1_ *_j_*; *C_j_* = 0 otherwise. *C* is the sum of the *C_j _*values for all species. It is the unscaled sum of *species gains* between T1 and T2.

The values *A*, *B,* and *C* are the building elements of the percentage difference, *D*_%diff_ = (*B* + *C*)/(2*A *+ *B* + *C*), and the Ružička dissimilarity, *D*_Ruz_ = (*B* + *C*)/(*A* + *B *+ *C*) (Podani et al., 2013; Legendre, 2014, Table S1.2). These two indices are interchangeable for TBI comparison although their values are not monotonically related. (*B* + *C*) represent the unscaled dissimilarity. The sign of (*C* – *B*) indicates the directionality of the process of losses and gains of individuals of the different species between the two surveys. *B* and *C* can be scaled by division by a denominator “den”, which is den_%diff_ = (2*A *+ *B* + *C*) for *D*_%diff_ index and den_Ruz_ = (*A* + *B *+ *C*) for the *D*_Ruz_ index. The *D*_%diff_ and *D*_Ruz_ dissimilarities measure the temporal beta diversity, or temporal change in community composition, for a site. The scaled *B* and *C* statistics can be called *D*_loss_ and *D*_gain_, where *D*_loss_ = *B*/den and *D*_gain_ = *C*/den. An interesting relationship is that *D*_loss_ + *D*_gain_ = *D*_%diff_ or *D*_Ruz_, depending on the denominator, den_%diff_ or den_Ruz_, that is used. In other words, *D*_loss_ and *D*_gain_ partition the *D*_%diff_ or *D*_Ruz_ dissimilarities into *loss* and *gain* components. Values of these indices are in the [0,1] range and are thus directly comparable.

The loss and gain statistics can be computed for occurrence (i.e., presence–absence) data as well, because *D*_%diff_ becomes the Sørensen dissimilarity with occurrence data and *D*_Ruz_ becomes the Jaccard dissimilarity, as mentioned above. The Sørensen and Jaccard dissimilarity equations for occurrence data are represented in book and paper equations with lower‐case instead of upper‐case letters (e.g., in Koleff et al., 2003, Legendre & Legendre, 2012, Legendre & De Cáceres, 2013); their equations, which exactly correspond to those of *D*_%diff_ and *D*_Ruz_, are *D*_Sørensen_ = (*b* + *c*)/(2*a *+ *b* + *c*) and *D*_Jaccard_ = (*b* + *c*)/(*a* + *b *+ *c*).

What are the ecological applications of *D*_loss_ and *D*_gain_? For each site, one can explore which process, between *D*_loss_ and *D*_gain_, shows the largest contribution to the temporal *D*_%diff_ or *D*_Ruz_ dissimilarity; in other words, which process is dominant at each site. The means of the *D*_loss_ and *D*_gain_ components across the sites express the dynamics of the community over all sites. For observations across a large number of sites within a region, or (e.g.) in all quadrats of a stem‐mapped dynamics forest plot, the *B*/den and *C*/den statistics can be mapped, plotted as species losses and gains in the new B–C plots described in Section 2.3 (see example in Ecological application 3), or studied in other ways (e.g., in Ecological application 2).

#### 2.1.2 Testing procedure

When it is pertinent to the problem at hand, TBI indices can be tested for significance. An example is found in Ecological application 1. The data are permuted at random in both matrices, as described below, and the index is recomputed. This procedure is repeated a large number of times, and a *p*‐value is calculated for the TBI difference between T1 and T2 at each site *i*. A detailed description of the permutation method follows.

**Figure 2:**
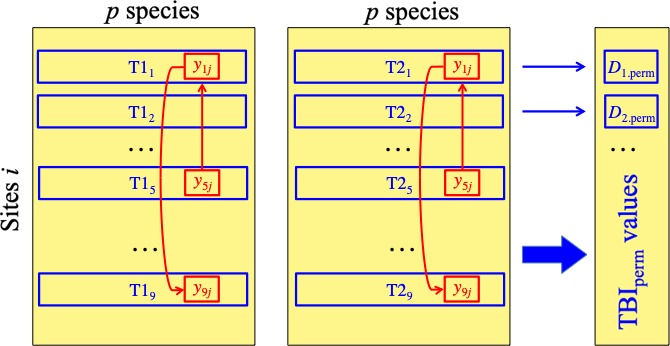
Random permutation of the community composition data is done separately for each species (column), in matrices T1 (left) and T2 (right). This figure shows an example where a permutation of species *j* brings value *y*_1_ *_j_* to position *y*_9_ *_j_* and value *y*_5_ *_j_* to position *y*_1_ *_j_*. The exact same permutation, involving all values in column *j*, is done in matrices T1 (left) and T2 (right). Following similar permutations of all *p* species, dissimilarities are computed between the two vectors representing each site *i*, producing the values *D_i_* under permutation

1. The null hypothesis (H_0_) of the TBI test is that a species assemblage at a focal site *i* (i.e., a site under study) is not exceptionally different between T1 and T2, compared to assemblages that could have been observed at the same two times at this site. The test involves a comparison of the TBI index computed for site *i* with other TBI indices obtained by randomization of the observed data under conditions corresponding to H_0_. The null hypothesis focuses on values of the TBI statistic, which is the dissimilarity between data vectors observed at times T1 and T2 for site *i*. The statistical decision (one‐tailed test) is taken as in any other parametric or permutational test; see paragraph 7 below.
2. Under the general hypothesis that the site data are permutable, the values in each column of **Mat.1** are permutable within that matrix, and similarly for **Mat.2**. The variation of each species among sites, in a given matrix, is assumed to be due to random sampling of a statistical population. Permutations are done for each species separately, following the concept that different species in an assemblage are under the influence of a variety of processes and do not form a pseudo‐organismic entity that would react as a unit to these varied processes. More about this in Note 1 at the end of the present subsection. Technical aspects of this permutation method are described in paragraph 3 underneath. Permutation of the species occurrence or abundance values in each column, independently of one another, was also the method used to assess the significance of the *Local Contributions to Beta Diversity* (LCBD indices, describing the contributions of individual sites) in the Legendre and De Cáceres (2013) paper. The same logic is followed here to test the significance of TBI indices, which are also indices about individual sites.
3. The data are permuted columnwise, species by species, and in exactly the same way in matrices T1 and T2 (Figure 2). To accomplish that in a computer function, a given permutation of the two matrices is started with the same random seed in both, and that seed is changed at the beginning of each new permutation. With this method, the abundance values of a given species at site *i* (e.g., site 1) in matrices T1 and T2 of the original data will be shifted to another site position (e.g., site 9) in both matrices in the permuted data. What is permuted is then a series of *differences* between T1 and T2, for each species separately.
4. In the TBI test, we are looking for site vectors whose dissimilarities between T1 and T2 would be exceptionally large. We are not interested in a systematic difference that would affect all sites concerning the loss or gain of a subset of the species, or of all species. Differences of this type are preserved, through the permutations, by *not permuting* data between **Mat.1** and **Mat.2**. Simulations reported in Supporting Information Appendix S1 showed that the test performed adequately in this respect; see Figures S1.2 and S1.4 and the associated tables of simulation results in that appendix.
5. If the selected TBI index is the chord, Hellinger, or log‐chord dissimilarity, a transformation is applied to the data before calculation of the Euclidean distance, as described in Section 2.1.1 of the Methods. For correct calculation of these indices under permutation, the transformation is recomputed on the permuted untransformed data matrices. This is necessary to make sure that the permuted data are transformed in the same way as the initial data, with row sums (for Hellinger) or row norms (for chord) being 1. In this way, the *D_i_* values of the permuted data remain comparable to the reference *D_i_*.
6. After permutation, the TBI distances between **Mat.1** and **Mat.2** are recomputed for all sites *i *separately.
7. After a large number of permutations, a p‐value is computed for each site *i*, as in any other permutation test. The test is one‐tailed in the upper tail since we are looking for sites with large TBI statistic values. Thus, a site that changed very little while the other sites changed a lot will not be found exceptionally different by this test, which was designed to identify sites where the change is greater than at most pseudo‐sites that can be produced by randomizing the data. If the one‐tailed *p*‐value is smaller than or equal to the significance level, for example, 0.05, H_0_ is rejected. Rejection may be due to two different situations that are both of interest to ecologists: (a) a TBI value in the upper tail of the statistical distribution may correspond to a site subjected to the same process as the other sites in the study, that site having produced a high TBI value by chance. Although this technically corresponds to a type I error, these extreme sites in the statistical distribution may still be interesting to examine. (b) Else, rejection may indicate a site where some special event has taken place between T1 and T2, different from what happened at other sites, causing a large difference to appear in community composition. Examples are climatic and geologic events, or the result of anthropogenic actions or processes. In both cases, the significant sites are of interest to ecologists who can concentrate work on them and investigate why community composition has changed in an exceptional way at these locations. Because the TBI indices for *n* sites are all tested at the same time, a correction for multiple testing must be applied to obtain a correct experiment‐wise error rate.

Note 1: *Ecological interpretation of the permutation models*—The permutation procedure described in paragraphs 2 and 3 above, whereby the values are permuted separately for each species, follows a concept of species assemblages that considers the species in an assemblage to be under the influence of a variety of processes; they do not form a pseudo‐organismic entity that would react as a unit to these processes. The first formulation of this concept is due to Gleason (1926) who argued that species responded individually to environmental conditions. He was opposing Clement's (1916) dominant view of the time that species assemblages formed an entity that reacted as a kind of pseudo‐organism. A permutation method based on Clement's pseudo‐organismic theory would permute at random entire rows of data in matrices **Mat.1** and **Mat.2**, not the values observed for each species separately. Most ecologists nowadays reject the pseudo‐organismic view of Clement and favor an alternative view, which is an extension of Gleason's, involving different ecological processes. The most important are environmental filtering (as in Gleason's theory), neutral processes which include ecological drift and limited dispersal (Hubbell, 2001), and interactions among species, all of which generate spatial structures in community data (Legendre, 1993; Legendre & Legendre, 2012). Permutation of values in individual species vectors, used in this paper and in the TBI.R function, follows this view about ecological communities.

Note 2—A different permutation method is used in tests of significance in linear statistical modeling (regression, RDA, CCA). In canonical analysis, this method consists in permuting entire rows of data in either the response or the explanatory matrix. This method implements Clement's view described in the previous paragraph. That permutation method was used in additional simulations. Results of these simulations are not reported in detail in Supporting Information Appendix S1 but they are summarized in the second paragraph of section “b. Permutation methods” in that Appendix.

Note 3—It is also possible to identify the species that have changed in a significant way between T1 and T2, either in the whole study area or in separate habitat types. This involves a different type of statistical test. The hypothesis (H_0_) that a species has not changed in abundances throughout the sites under study can be tested using a paired *t* test. Because species abundances are not normally distributed, a permutation test must be used. The permutations involve the two facing values of the species observed at each site at T1 and T2. A correction for multiple testing must be applied to the *p*‐values because several species are tested simultaneously. This method was used in the Legendre and Condit (2019) companion paper. An R function implementing that test is available; see Section 2.4, first paragraph.

### 2.2 Partitioning dissimilarities into losses and gains

When the percentage difference or the Ružička dissimilarity are used as TBI indices, *B* is the unscaled sum of *species losses* and *C* is the unscaled sum of *species gains* between T1 and T2. These statistics are described in Section 2.1.1 above. The unscaled statistics can be scaled to the [0,1] range by division by the percentage difference denominator den = (2*A *+ *B* + *C*) or by the Ružička denominator den = (*A* + *B *+ *C*). The *dissimilarity* *D* is (*B*/den + C/den) = (*B* + *C*)/den. If the TBI dissimilarity is either the percentage difference or the Ružička dissimilarity, one can take advantage of this decomposition of *D* and list the *B*/den and *C*/den components of TBI indices for each site in the study. Examples are shown in Appendices S3 and S4, which complement Ecological applications 1 and 3. These basic statistics can be used in two different ways:

1. We can compute summary statistics: the mean of (*B*/den), the mean of (*C*/den), and the mean of *D* = (*B* + *C*)/den. The following relationship holds: mean(*B*/den) + mean(*C*/den) = mean(*D*). From this decomposition of *D*, we can derive the contribution of the species losses to the total dissimilarity, *B*/(*B* + *C*), and similarly the contribution of the species gains to the total dissimilarity, *C*/(*B* + *C*). These two ratios sum to 1, providing the relative importance of the species losses and gains phenomena. The result is the same for calculation without a denominator den, or with either the percentage difference or the Ružička denominator.
2. For each site, we can also obtain the sign of the difference (gains–losses) or (*C*–*B*): if *C* > *B,* we note a plus (+) sign, and if *C* < *B* we note a minus sign (–) in the function output list. This notation allows users to quickly identify the sites where gains or losses dominate. Similarly, the difference mean(*C*/den)–mean(*B*/den) is computed; its sign tells us if gains (+ sign) or losses (– sign) dominate across all sites. The significance of the difference between the two vectors of statistics *B*/den and *C*/den can be computed using a parametric or permutational paired *t* test; the TBI function mentioned in *Software* (Section 2.4) computes both forms. These tests provide overall indications of the direction of change in community composition over all sites. They help confirm the asymmetry between abundance or occurrence losses (*B*/den) and abundance or occurrence gains (*C*/den). In Ecological application 2 (Tikus Island coral communities), the two forms of calculation, on abundance and occurrence data, provided complementary information.

### 2.3 Species losses and gains: the B–C plot

We can also use the *B*/den and *C*/den statistics as coordinates of points (representing sites) in bivariate graphs with *B*/den in the abscissa and *C*/den in the ordinate. We call these graphs *B–C plots*. They display visually the relative importance of the loss and gain processes across the study sites, informing researchers about the detailed and global structure of the species losses and gains.

A B–C plot is presented in Ecological application 3 (Chesapeake Bay benthos data). In that B–C plot, a diagonal green line, with slope of 1, was drawn through the origin; it represents the theoretical positions of sites where *D*_gain_ would be equal to *D*_loss_. A red line was also drawn parallel to the green line, passing through the centroid of all points. When the red line is below the green line, it indicates that the survey interval was dominated by species losses across the sites, and the opposite if the red line is above the green line. Points found higher in the plot, in the diagonal direction toward the upper‐right corner, represent higher temporal beta diversity than points found lower in the direction of the lower‐left corner.

In B–C plots, the points representing sites can be labeled with colors or symbols representing the types of environment, the geographic areas where they come from, or any other independent classifier of interest. Separate B–C plots can be drawn for sites surveyed in different types of environment, although all sites may have been analyzed in the same TBI analysis. Comparison of these separate plots will immediately show which types of environment have produced mostly losses or gains in species occurrences or abundances.

### 2.4 Software

These calculations are implemented in the *TBI.R* function available in the R package *adespatial* (Dray et al., 2019). Function plot.TBI.R is also available in *adespatial* to draw B–C plots. Examples of output files of the TBI function are shown in Appendices S3 and S4, which complement Ecological applications 1 and 3. Function tpaired.krandtest.R can be used to identify the species whose abundances have changed in a significant way between T1 and T2. That function is described in Supporting Information Appendix S5; it is also available in the R package *adespatial* on CRAN.

Another R package, *codyn* (Hallett et al., 2018, 2016), computes a variety of metrics for temporal diversity analysis. The percentage difference *D* is used as one of the metrics for temporal comparison of communities observed at different times at a focal site. Function *turnover* of that package includes an option to compute species losses and gains divided by the %difference denominator, as in the *TBI.R* function; losses and gains are the indices called *B* and *C* in the present paper.

## 3 NUMERICAL SIMULATIONS

Numerical simulations were used to check the type I error rate and power of the permutation method described in Section 2.1.2 above. The data simulation methods and results are described in detail in Supporting Information Appendix S1. A summary of these results is presented here, with recommendations to users.

### 3.1 Simulation to estimate type I error rates

The simulation results reported in Supporting Information Appendix S1 show that the TBI tests had correct rates of type I error for the two community‐like data generation methods (Poisson and lognormal) and all dissimilarity indices available in the *TBI.R* function, and this for all significance levels (*α*) considered, from *α* = 0.01 to *α* = 0.50. The testing method is thus valid in all these circumstances (Edgington, 1995).

### 3.2 Simulations to compare power of *D* indices

For the analysis of community composition data, the percentage difference and Ružička indices produced tests with the highest power, followed by the indices in the Box–Cox family: the chord, Hellinger, and log‐chord distances. The Euclidean distance alone produced TBI tests with extremely low power with community composition‐like data. This distance should not be used for TBI tests of community composition data (Figures S1.5 and S1.6, Supporting Information Appendix S1). However, power was high enough to recommend the Euclidean distance for test involving standardized environmental data.

In summary, the best combination to obtain TBI tests of community data with maximum power is to use the percentage difference or the Ružička indices. These two dissimilarities can also be decomposed into species losses (*B*/den) and gains (*C*/den), which can be used to examine the processes of losses and gains at the site level and produce B–C plots.

Additional simulations involving different numbers of sites with an effect and different total numbers of sites showed that power of the test was high as long as the proportion of sites with an effect to be detected (i.e., sites made to be exceptional) was smaller than *n*/2, where *n* is the total number of sites in the study (Figure S1.7, Supporting Information Appendix S1).

## 4 TBI ANALYSIS OF ENVIRONMENTAL OR TRAIT DATA

It could be interesting to identify the sites where the changes in environmental conditions (e.g., land use) were the most important. The TBI method can be used to compare two matrices containing the same environmental variables observed at T1 and T2 and determine, for example, if these sites are also those where the community has changed the most. The analysis of environmental variables is a situation where the Euclidean distance would be appropriate as a basis for computing a TBI index.

- If all environmental variables are quantitative, they should be standardized using the same parameters (means, standard deviations) for matrices T1 and T2, before they are used as input in TBI analysis. How to do that is described and implemented in a function provided in Supporting Information Appendix S2.
- If the data are factors or a mix of quantitative and factor variables, one should join matrices T1 and T2 one on top of the other, as described in the explanation paragraph of Supporting Information Appendix S2, and then compute a Gower dissimilarity matrix **D**. Apply principal coordinate analysis (PCoA) to the square‐rooted Gower dissimilarities because a Gower **D** matrix is non‐Euclidean. Square rooting should make the Gower matrix Euclidean, which is necessary before PCoA in this case; we have to recuperate and use all PCoA axes for TBI analysis; hence, the values should be real and not complex numbers. Then, separate the two transformed matrices and use them as input into TBI analysis.
- For community trait matrices with mixed precision levels (quantitative and qualitative traits), use the same method as in the previous paragraph: compute a Gower dissimilarity matrix, as recommended by Laliberté and Legendre (2010), then PCoA of the square‐rooted dissimilarities; split the principal coordinates into two matrices and compute TBI analysis using the Euclidean distance. No application of TBI analysis to environmental or trait data is presented in this paper to save space.

## 5 ECOLOGICAL APPLICATIONS

The three ecological applications that follow use multivariate community data. They were chosen to illustrate different facets of TBI analysis, not to draw ecological conclusions about these three particular ecosystems. Application 1 illustrates the importance of carrying out TBI analysis using a dissimilarity index designed for the analysis of community composition data; the Euclidean distance produced nonsignificant and uninterpretable results. In application 2, The *B* and *C* components of the TBI dissimilarity are used to analyse the effects of a major disturbance (El Niño) on communities; the analysis is complemented with a standard canonical analysis of the community data. Application 3 illustrates the construction and interpretation of a B–C plot.

### 5.1 Ecological application 1—Insecticide treatments in mesocosms

Observations on the abundances of 178 invertebrate species (macroinvertebrates and zooplankton) subjected to insecticide treatments in aquatic mesocosms (called “ditches”) were used by van den Brink and ter Braak (1999) as an application example in their paper describing Principal Response Curves (PRC) analysis. The authors agreed to make the data available to researchers in the CANOCO program documentation and in the R package vegan (Oksanen et al., 2017).

The experiment involved twelve mesocosms, which were surveyed on eleven occasions. Four mesocosms served as controls (dose = 0) and the remaining eight were treated once with the insecticide chlorpyrifos, with dose levels of 0.1, 0.9, 6.0, and 44.0 μg/L in two mesocosms each. The data are log‐transformed species abundances, *y*_tr_ = log_e_(10*y* + 1). In their paper, the authors used the log‐transformed invertebrate data in PRC analysis; PRC preserved the Euclidean distance among the observations.

The 12 mesocosms had been attributed at random to the treatments. However, to facilitate presentation of the results, they will be presented here in order of increased insecticide doses: {0, 0, 0, 0, 0.1, 0.1, 0.9, 0.9, 6.0, 6.0, 44.0, 44.0} μg/L.

Results of the calculations with the R function TBI() are presented for the species abundance and occurrence data of this ecological application. We will compare data of surveys #4 and #11. Survey #4 was done one week after the insecticide treatment in the mesocosms, and the fauna was considered to have fully recovered from treatment at the time of survey #11. To give examples, in the two mesocosms that had received the highest insecticide doses, species richness increased by 9 and 19 species from survey #4 to #11.

The TBI dissimilarities showed that in the two mesocosms with the highest insecticide doses, community compositions were the most different between T1 and T2 (Table 1, upper panel). The test found the changes in these two communities to be exceptional with reference to the T1–T2 difference found in communities simulated by permutations of the data obtained during in the experiment. The two mesocosms that had received the highest doses of the insecticide, M11 and M12, showed exceptional differences in community composition for the percentage difference and Ružička dissimilarities (Table 1, lower panel). The chord, Hellinger, and log‐chord distances led to the same conclusion. These five distances had been deemed appropriate for beta‐diversity study (Legendre & De Cáceres, 2013). On the contrary, the Euclidean distance is known to be inappropriate for such studies (Legendre & Legendre, 2012; Orlóci, 1978) and, indeed, TBI tests based on that distance did not produce significant differences in community composition between surveys #4 and #11 in any of the mesocosms, including M11 and M12.

Detailed analysis of the species losses (*B/*den) and gains (*C/*den), obtained from TBI analysis computed with the percentage difference (Supporting Information Appendix S3, first section), showed that in the eight treated mesocosms (called Sites 5 to 12 in Supporting Information Appendix S3), the changes in community composition (abundance data) always consisted of species gains; that is, statistic *C/*den (gains) was always larger than *B/*den (losses). The mean values of *B/*den and *C/*den for these eight mesocosms showed that gains (*C/*den) represented 56% of the dissimilarities, as expected in a study of recovery after an insecticide treatment. The permutational paired *t* test showed a highly significant difference (*p* = 0.0066) between losses and gains across the eight treated mesocosms.

TBI calculations using the Sørensen *D* (Supporting Information Appendix S3, Section 2) indicated that, in addition to mesocosms #11 and 12, mesocosm #10, treated with 6 μg/L of insecticide, also displayed a significant difference between T1 and T2 at significance level 0.05. This result indicates that in the insecticide experiment, the reappearance of species (positive change) gave a clearer signal of community recovery than the increase in species abundances (Supporting Information Appendix S3, Section 1).

### 5.2 Ecological application 2—South Tikus Island coral communities

Brown and Suharsono (1990) surveyed coral communities (75 species) at 10 sites in the island of South Tikus, Indonesia, in the years 1981, 1983, 1984, 1985, 1987, and 1988. An El Niño event occurred in 1982–1983, which caused coral bleaching and death of coral colonies, and triggered changes in the composition of coral communities. They reported that “as many as 80–90% of corals died on the reef flats at the study sites, with the major casualties being branching species in the genera *Acropora* and *Pocillopora*.”

Coral forms colonies which occupy surfaces, so that the data are not in numbers of individuals but in areal cover of each species. The sum of the species areal covers at a site may exceed 100% because coral colonies may overlap one another vertically. The Brown and Suharsono (1990) data have been used in several papers to demonstrate the application of multivariate methods for the analysis of beta diversity and the comparison of surveys across time, for example, by Warwick, Clarke, and Suharsono, (1990), Anderson et al. (2011), and Chao and Chiu (2016). Following these papers, the data in the present application were treated as if they were species abundances. They were obtained from Appendix S1 of the Anderson et al. (2011) article.

**Figure 3:**
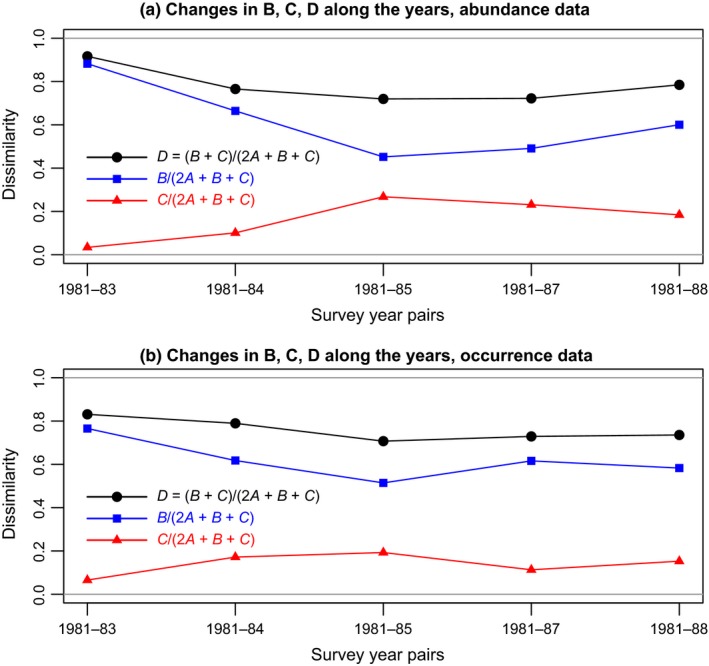
Tikus Island coral data. (a) Changes in dissimilarity *D* computed from the quantitative coral community compositions between years, and its components *B*/den (losses) and *C*/den (gains); den is the denominator of the dissimilarity index *D*, (2*A *+ *B* + *C*) in this figure. The 1981 survey, before the El Niño event, is compared in turn to the 1983, 1984, 1985, 1987, and 1988 surveys. (b) Same for the species occurrence (i.e., presence–absence) data

**Figure 4:**
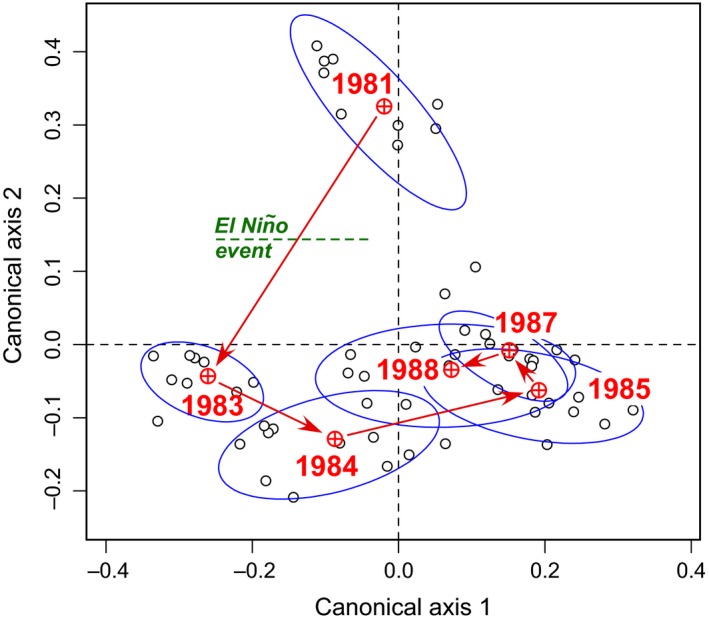
Tikus Island coral data. Canonical ordination plot obtained by dbRDA for the quantitative coral community compositions data for the 6 years and 10 sites, constrained by a factor representing the 6 survey years. The years are marked by red symbols, and the sites (open circles) for each year are incompletely surrounded by 60% coverage ellipses. Arrows materialize the sequence of years

The data were analyzed in two complementary ways. (a) Figure 3 presents an analysis of the species loss (*B*/den) and gain (*C*/den) components of the dissimilarity *D* between the 1981 survey, before the El Niño event, and the five following surveys: 1983, 1984, 1985, 1987, and 1988, for abundance and presence–absence data. (b) This analysis is completed with a redundancy analysis (RDA) biplot shown in Figure 4, showing the changes in community composition with time. This biplot was produced as follows: first, a percentage difference matrix was computed among all years and sites; the dissimilarities were square‐rooted to make the matrix Euclidean, and that matrix was subjected to principal coordinate analysis (Gower, 1966). The entire matrix of principal coordinates was used as the response data in a RDA against a factor representing the six survey years of the study. This form of canonical ordination is called distance‐based redundancy analysis (dbRDA, Legendre & Anderson, 1999).

We will examine the changes in community composition between the 1981 survey, before the El Niño event, and the following five surveys: 1983, 1984, 1985, 1987, and 1988. This study is not meant to identify sites that were exceptionally different between two years or test specific hypotheses about them. Instead of testing the TBI statistics for sites, we will carry out a detailed study of the species loss (*B*/den) and gain (*C*/den) statistics described in the Methods. These statistics were computed with the denominator (den) of the percentage difference index, *D*_%diff_; they decompose the percentage difference *D* into additive components, losses (*B*/den) and gains (*C*/den).

First, we will plot the mean values of the *B*/den, *C*/den*,* and *D* statistics computed across the sites, in comparisons with the 1981 survey (before the El Niño event) with all successive surveys in turn (1983, 1984, 1985, 1987, and 1988, after El Niño) (Figure 3), in order to study the effect of the El Niño event on the communities. This method of analysis had been used by Legendre and Salvat (2015, Figure 3), who described the effects of a nuclear test on the mollusk communities of an atoll in the Pacific.

Figure 3a shows the changes in *D* between years, and its components *B*/den and *C*/den. We observe that after El Niño, species losses (*B*/den) dominated the changes, accounting for 96% of the mean dissimilarity (*D*) between 1981 and 1983; species gains (*C*/den) represented only 4% of mean *D*. In later years, the species losses decreased whereas gains increased till the 1981–1985 comparison. The TBI function was run over all year pairs over the 10 study sites. Results showed that dominance of *B/*den (losses) over *C*/den (gains) was significant for all year pairs, as shown by the overall paired *t* tests of the asymmetry, described in the Section 2.

Does that mean that some of the species that had disappeared had recovered, or that only the species that remained had increased their abundances‐per‐species? The answer is found in Figure 3b, which displays the same statistics computed for species occurrence data. In that graph, the *B*/den and *C*/den lines would be horizontal at value 0 if all species had remained present and the change after El Niño was only in the abundances. Instead, that graph shows that many species disappeared at first from the surveyed sites after El Niño (*B*/den was 0.77 for the 1981–1983 comparison). Then some of the original species recovered on the reefs (*B*/den decreased to 0.62 for 1981–1984 and to 0.51 for 1981–1985), possibly by budding from colonies that had survived at nearby sites or by dispersion of larvae from elsewhere. Species losses stabilized around 0.60 in later years compared to the 1981 community composition. During that time, new species that were not present in 1981 occupied the depleted reefs, starting in the 1981–1983 comparison (*C*/den = 0.06) and increasing in the following years (0.17 for 1981–1984 and 0.19 for 1981–1985). Gains of new species, compared to 1981, stabilized around 0.10–0.15 in later years.

Whereas the dissimilarity values *D* remained large for species abundance and occurrence data, the *changes* in *D* became small in the later‐year comparisons and were possibly caused by sampling variation. The large values of *D* between 1981 and the post‐El Niño years showed that the coral communities had settled to a new composition that was very different from what it was in 1981: from 1981–1984 to 1981–1988, *D* remained around 0.75 for the abundance data (Figure 3a) and 0.74 for the presence–absence data (Figure 3b).

The overall similarity in community composition between years can be appreciated in a RDA biplot, where the centroid of each year is shown surrounded by the 10 site observations of that year (Figure 4). Computation of the biplot is described in the third paragraph of the present section. The figure shows that the sites in 1981 had quite different species composition than in surveys after El Niño. In 1983, the communities moved to a position in the ordination very distant from 1981 after heavy species losses; then it moved to a new position in 1984 after it recuperated some of its former species, plus some new species that were not present in 1981 and 1983. It moved again in 1985. From then on, the changes observed in 1987 and 1988 seem to represent random variation due to observed random losses and gains of species, which may be due in part to sampling variation and in part to random species losses and gains.

The communities found in South Tikus Island after the natural El Niño event strongly differed in species composition from the structure they had in 1981, and they kept changing, apparently randomly, in later years. A similar phenomenon was shown in the Legendre and Salvat (2015) study, where the disturbance of marine mollusk communities was due to a strong man‐made disturbance. In both cases, the observed changes are compatible with the neutral theory of generation of biodiversity and changes in communities (Hubbell, 2001).

### 5.3 Ecological application 3—Chesapeake Bay data

The data set used in this example was extracted from the Maryland Data Sets of the Chesapeake Bay Benthic Monitoring Program (http://www.baybenthos.versar.com/data.htm), which is a portion of the Chesapeake Bay Program (http://www.chesapeakebay.net/). Detailed information about the sampling protocol is found on that web page. The data, available online, come in the form of numerous text files, one per group of variables and per year. Legendre and Gauthier (2014) compiled and formatted the separate data files in a *Rdata* file for immediate analysis in R. The <ChesapeakeBay.Maryland.RData> data are available in a zipped file found in Appendix S5 of their paper. The file contains macrofaunal data (203 invertebrates and 2 chordates) collected in the sediment of 27 sites of the bay, spring, and fall, during 13 years, that is, from 1996 to 2008, for a total of 702 data rows.

Table 2 shows how the species are split between seasons and salinity groups. The spring survey data contain 181 species and the fall data 142 species. Two freshwater sites (#36 and #79) were present in the database; they contained 105 species. These two sites were excluded from the present example, which focuses on the 25 brackish sites where 155 species were identified. During the fall surveys in 2005 and 2008, 52 species (abundance data) were observed: 38 in 2005 and 45 in 2008, with an intersection of 31 species found in both years.

**Table 2:** Number of species in subsets of the Chesapeake Bay fauna data surveyed during 13 years, spring and fall. In total, 205 benthic species were found at the 27 survey sites

	Spring	Fall	Spring and fall
Freshwater (two sites)	93	58	105
Brackish (25 sites)	128	121	155
All survey sites (27 sites)	181	142	205

**Figure 5:**
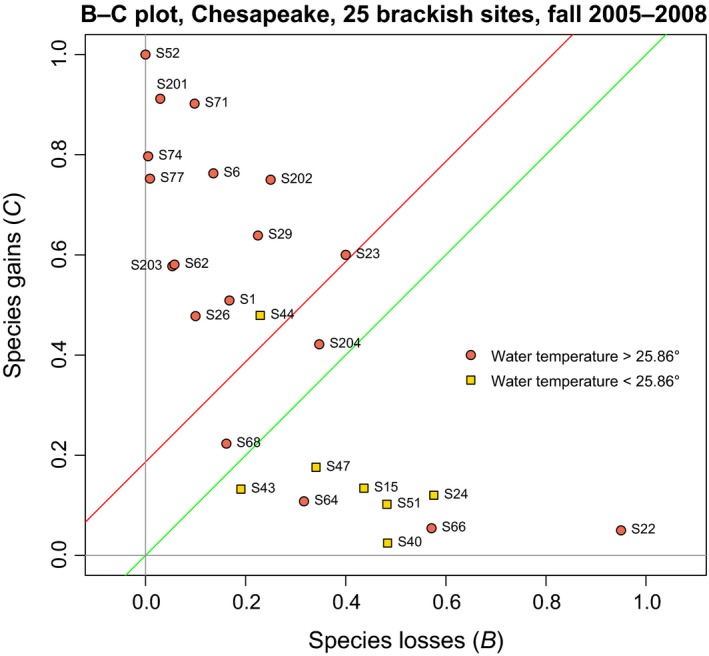
Chesapeake Bay benthos data. B–C plot comparing the fall surveys of 2005 and 2008, where the 25 brackish sites are plotted using the losses (*B/*den statistics) and gains (*C/*den statistics) computed from the species abundance data. Sites are identified by their code of the Chesapeake Bay Benthic Monitoring Program. The sites are represented by symbols corresponding to two water temperature groups observed during the 2005 fall survey. Green line with slope of 1: line where gains equal losses. The red line was drawn parallel to the green line (i.e., with slope = 1) and passing through the centroid of the points. Its position above the green line indicates that, on average, species gains dominated losses from 2005 to 2008

This example offers the opportunity to build a B–C plot described in Section 2.3 of the Methods. The percentage difference index was used; the Ružička index would have produced similar results. These data will be used to demonstrate how to draw a B–C plot and how to interpret it. For the year pair 2005 and 2008, the B–C plot is shown in Figure 5. In the plot, the red line is *above *the green line. This indicates that *gains* in benthic abundances‐per‐species dominated *losses* in the Chesapeake brackish sites (fall surveys) from 2005 to 2008.

A simple classification of the sites by an environmental factor, water temperature during the 2005 fall survey, was used to separate the sites in two groups, providing an example of the kind of information that can be derived from displaying different habitat groups as symbols or colors in B–C plots. These two groups of sites could also be drawn in separate B–C plots. These separate plots would show that species losses dominated in the warmer sites, whereas species gains dominated in the colder sites. The B–C plot is an appropriate tool to display this ecological relationship.

**Figure 6:**
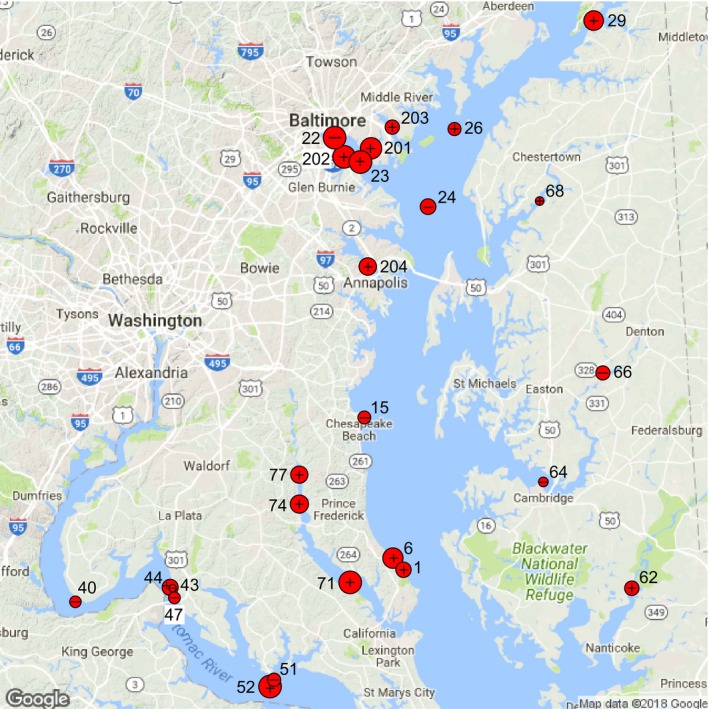
Map of the 25 brackish sites (red symbols) of the Chesapeake Bay ecological survey produced with the RgoogleMaps package in R. Comparison of surveys in years 2005 and 2008, abundance data: point sizes are proportional to the TBI indices (percentage difference *D*). + signs indicate the 17 sites where *gains* in abundances‐per‐species dominated; – signs, the 8 sites where *losses* dominated. The site identification numbers are those found in the Chesapeake Bay data base

In addition to the computation of the *B*/den and *C*/den components at each site, the R function also computed TBI indices (Supporting Information Appendix S4). A map of the 25 brackish sites on the Chesapeake Bay, plotted with the RgoogleMaps package, is shown in Figure 6. On the map, symbol sizes are proportional to the TBI indices and signs on the symbols indicate the sites dominated by abundance‐per‐species gains (+) and losses (–) between 2005 and 2008.

## 6 CONCLUDING REMARKS AND PROSPECTIVE APPLICATIONS

TBI analysis and B–C plots are useful to identify exceptional sites in space–time ecological surveys carried out to study the effects of natural and anthropogenic changes to ecosystems. Such studies are presently carried out by teams of ecologists around the world. They are collecting data over land, in lakes and in the oceans to assess the effects of climate change on natural communities and other types of biodiversity data. Researchers would like to identify the sites where important changes have taken place. They can then focus their attention onto these sites and seek what has been going on there, and why community composition has changed in an exceptional way at these sites. The TBI method was designed for this type of research.

The present paper is the first description of the ecological theory and statistical developments behind TBI analysis, describing the method in its present state of development. It should provide opportunities to researchers to apply this new method of analysis to a broad palette of ecological and genetic questions.

The paper has shown that it is possible to compute a valid test of significance for dissimilarity indices, which are used to compare data about sites collected at different times. Additionally, the paper has shown how four of the TBI indices can be decomposed into loss and gain components (of species, or abundances‐per‐species) and how these components can be used to produce B–C plot, a new type of plot that informs users about the processes of biodiversity losses and gains through time found in space–time survey data.

The following indices were found to be appropriate for computation and testing of TBI indices for community composition data: the percentage difference, Ružička, Sørensen, Jaccard, chord, Hellinger, and log‐chord indices. The first four present the advantage that they can be decomposed into species losses and gains at each site, which makes them the preferred choices in most studies. In studies where community data must be projected into Euclidean space, for instance before PCA, RDA, or other forms of linear analysis, data can be transformed using the chord, Hellinger, and log‐chord transformations, and the TBI analysis can be performed using the corresponding *D* indices, that is, the chord, Hellinger, or log‐chord distances. The simulation study has also shown that the Euclidean distance is inappropriate for TBI analysis of community composition data. It can be used, however, for TBI analysis of environmental or species trait data.

The *B* (species losses) and *C* (gains) components are also the building blocks of the numerators of the replacement and nestedness or richness/abundance difference indices of Andrés Baselga (Baselga, 2010, 2012) and János Podani (Podani et al., 2013; Podani & Schmera, 2011). These indices were reviewed by Legendre (2014). These authors have shown that the Jaccard/Sørensen and Ružička/percentage difference *D* indices can be decomposed into replacement (or spatial turnover), 2 min(*B*,*C*) and richness/abundance difference, |*B*–*C*|, for either presence–absence or abundance data. Because they developed their indices for spatial analysis, Baselga and Podani did not emphasize the asymmetry implicit in a temporal comparison between T1 and T2. In temporal studies, where processes are directional, comparison of the loss (*B*) and gain (*C*) components, which are central to the TBI method described in the present paper, is informative.

Analysis of the *B* and *C* components brings us to the heart of the mechanisms by which communities change through time: losses (*b*) and gains (*c*) of species, losses (*B*) and gains (*C*) of individuals of the various species. B–C analysis is especially interesting in species‐rich communities where researchers cannot examine the changes in each species individually.

B–C analysis can also be applied to subgroups of sites, for example, habitat types. In addition, it can be used to compare the changes that occurred in specific groups of species that are known to react differently to environmental stressors, for example, different age or size classes, or species of different origins, for example: the temperate, transitional, and boreal trees found together in the forested southern portion of Canada.

Different ecological applications were worked out with co‐authors during the development of the TBI method. Some of them have already been published. Working on these papers provided opportunities to develop the TBI theory and software, through the analysis of pertinent application questions, hypotheses, and data. These applications provide examples that ecologists may find useful as guides for the analysis of their own data, in addition to the ecological applications summarized in the previous section of the paper:

- Impact of a field experiment—The loss (*B*/den) and gain (*C*/den) statistics were first analyzed by Legendre and Salvat (2015) to compare community composition data (marine mollusks) during 30 years, before and after a man‐made disturbance on an atoll in the Pacific. This disturbance to the mollusk community was the atmospheric test of a Hydrogen bomb in 1968.
- A paleoecological study—Winegardner et al. (2017) compared diatom communities in lake sediment surveyed 150 years apart across the USA. Temporal beta‐diversity varied significantly as a function of forest cover, with higher temporal beta in watersheds with contemporary lower forest cover.
- Space–time freshwater ecology—Kuczynski, Legendre, and Grenouillet (2018) compared freshwater fish surveys 20 years apart in rivers throughout France. They observed biotic homogenization over time in fish communities. Changes in community composition mainly resulted from population declines and were favored by an increase in temperature seasonality and in non‐native species density.
- Forest ecology—Legendre and Condit (2019) analyzed B–C plots for six habitat types, comparing tree community composition (abundance data) from the surveys conducted 30 years apart, in 1985 and in 2015, in the Barro Colorado Island Forest Dynamics Plot in Panama (50 ha) divided into 1,250 (20 m × 20 m) quadrats.

In a particular study, researchers may be mostly interested in identifying the sites with high and significant TBI indices. In other studies, interest may be in a fine analysis of the changes in the loss and gain components of the dissimilarity in community composition, compared to a pre‐disturbance situation. One can look at these components in graphs that allow researchers to compare, for example, different subsets of the data. Ecological examples have been shown in the paper for these different situations.

## CONFLICT OF INTEREST

None declared.

## AUTHOR CONTRIBUTIONS

P. Legendre designed the methods described in this paper, programmed the software, conducted the analyses of the numerical examples and the simulation study, and wrote the manuscript.

## Data Availability

The data used in the ecological applications are publicly available in the references provided.

## References

[ece34984-bib-0001] Anderson, M. J. (2001). Permutation tests for univariate or multivariate analysis of variance and regression. Canadian Journal of Fisheries and Aquatic Sciences, 58, 626–639. 10.1139/f01-004

[ece34984-bib-0002] Anderson, M. J. (2006). Distance‐based tests for homogeneity of multivariate dispersions. Biometrics, 62, 245–253. 10.1111/j.1541-0420.2005.00440.x 16542252

[ece34984-bib-0003] Anderson, M. J. , Crist, T. O. , Chase, J. M. , Vellend, M. , Inouye, B. D. , Freestone, A. L. , … Swenson, N. G. (2011). Navigating the multiple meanings of β diversity: A roadmap for the practicing ecologist. Ecology Letters, 14, 19–28. 21070562 10.1111/j.1461-0248.2010.01552.x

[ece34984-bib-0004] Angeler, D. G. , Viedma, O. , & Moreno, J. M. (2009). Statistical performance and information content of time lag analysis and redundancy analysis in time series modeling. Ecology, 90, 3245–3257. 10.1890/07-0391.1 19967879

[ece34984-bib-0005] Baselga, A. (2010). Partitioning the turnover and nestedness components of beta diversity. Global Ecology and Biogeography, 19, 134–143. 10.1111/j.1466-8238.2009.00490.x

[ece34984-bib-0006] Baselga, A. (2012). The relationship between species replacement, dissimilarity derived from nestedness, and nestedness. Global Ecology and Biogeography, 21, 1223–1232. 10.1111/j.1466-8238.2011.00756.x

[ece34984-bib-0007] Brown, B. E. , & Suharsono . (1990). Damage and recovery of coral reefs affected by El Niño related seawater warming, in the Thousand Islands, Indonesia. Coral Reefs, 8, 163–170.

[ece34984-bib-0008] Chao, A. , & Chiu, C.‐H. (2016). Bridging the variance and diversity decomposition approaches to beta diversity via similarity and differentiation measures. Methods in Ecology and Evolution, 7, 919–928. 10.1111/2041-210X.12551

[ece34984-bib-0009] Clements, F. E. (1916). Plant succession: An analysis of the development of vegetation. Washington, DC: Carnegie Institution of Washington.

[ece34984-bib-0010] Dray, S. , Bauman, D. , Blanchet, G. , Borcard, D. , Clappe, S. , Guénard, G. , … Wagner, H. H. (2019) adespatial: Multivariate multiscale spatial analysis. R package version 0.3‐3. Retrieved from https://cran.r-project.org/package=adespatial

[ece34984-bib-0011] Edgington, E. S. (1995). Randomization tests, 3rd ed. New York, NY: Marcel Dekker.

[ece34984-bib-0012] Gleason, H. A. (1926). The individualistic concept of the plant association. Bulletin of the Torrey Botanical Club, 53, 7–26. 10.2307/2479933

[ece34984-bib-0013] Gower, J. C. (1966). Some distance properties of latent root and vector methods used in multivariate analysis. Biometrika, 53, 325–338. 10.1093/biomet/53.3-4.325

[ece34984-bib-0014] Hallett, L. M. , Avolio, M. H. , Carroll, I. T. , Jones, S. K. , MacDonald, A. A. M. , Flynn, D. F. B. , … Jones, M. B. (2018). codyn: Community dynamics metrics. R Package Version, 2.0. Retrieved from https://github.com/NCEAS/codyn

[ece34984-bib-0015] Hallett, L. M. , Jones, S. K. , MacDonald, A. A. M. , Jones, M. B. , Flynn, D. F. B. , Ripplinger, J. , … Collins, S. L. (2016). codyn: An R package of community dynamics metrics. Methods in Ecology and Evolution, 7, 1146–1151.

[ece34984-bib-0016] Hubbell, S. P. (2001). The unified neutral theory of biodiversity and biogeography. Princeton, NJ: Princeton University Press.

[ece34984-bib-0017] Koleff, P. , Gaston, K. J. , & Lennon, J. J. (2003). Measuring beta diversity for presence‐absence data. Journal of Animal Ecology, 72, 367–382. 10.1046/j.1365-2656.2003.00710.x

[ece34984-bib-0018] Kuczynski, L. , Legendre, P. , & Grenouillet, G. (2018). Concomitant impacts of climate change, fragmentation and non‐native species have led to reorganization of fish communities since the 1980s. Global Ecology and Biogeography, 27, 213–222. 10.1111/geb.12690

[ece34984-bib-0019] Laliberté, E. , & Legendre, P. (2010). A distance‐based framework for measuring functional diversity from multiple traits. Ecology, 91, 299–305. 10.1890/08-2244.1 20380219

[ece34984-bib-0020] Legendre, P. (1993). Spatial autocorrelation: Trouble or new paradigm? Ecology, 74, 1659–1673.

[ece34984-bib-0021] Legendre, P. (2014). Interpreting the replacement and richness difference components of beta diversity. Global Ecology and Biogeography, 23, 1324–1334. 10.1111/geb.12207

[ece34984-bib-0022] Legendre, P. , & Anderson, M. J. (1999). Distance‐based redundancy analysis: Testing multispecies responses in multifactorial ecological experiments. Ecological Monographs, 69, 1–24. 10.1890/0012-9615(1999)069[0001:DBRATM]2.0.CO;2

[ece34984-bib-0023] Legendre, P. , & Borcard, D. (2018). Box‐Cox‐chord transformations for community composition data prior to beta diversity analysis. Ecography, 41, 1–5. 10.1111/ecog.03498

[ece34984-bib-0024] Legendre, P. , & Condit, R. (2019). Spatial and temporal analysis of beta diversity in the Barro Colorado Island forest dynamics plot, Panama. Forest Ecosystems. 10.1186/s40663-019-0164-4

[ece34984-bib-0026] Legendre, P. , & De Cáceres, M. (2013). Beta diversity as the variance of community data: Dissimilarity coefficients and partitioning. Ecology Letters, 16, 951–963. 10.1111/ele.12141 23809147

[ece34984-bib-0027] Legendre, P. , De Cáceres, M. , & Borcard, D. (2010). Community surveys through space and time: Testing the space‐time interaction in the absence of replication. Ecology, 91, 262–272. 10.1890/09-0199.1 20380215

[ece34984-bib-0028] Legendre, P. , & Gauthier, O. (2014). Statistical methods for temporal and space‐time analysis of community composition data. Proceedings of the Royal Society B: Biological Sciences, 281, 20132728 10.1098/rspb.2013.2728 PMC3906937 24430848

[ece34984-bib-0029] Legendre, P. , & Legendre, L. (2012). Numerical ecology, 3rd English edition. Amsterdam, The Netherlands: Elsevier Science BV.

[ece34984-bib-0030] Legendre, P. , & Salvat, B. (2015). Thirty‐year recovery of mollusc communities after nuclear experimentations on Fangataufa atoll (Tuamotu, French Polynesia). Proceedings of the Royal Society B: Biological Sciences, 282, 20150750 10.1098/rspb.2015.0750 PMC4590484 26063849

[ece34984-bib-0031] McEwan, R. W. , Dyer, J. M. , & Pederson, N. (2011). Multiple interacting ecosystem drivers: Toward an encompassing hypothesis of oak forest dynamics across eastern North America. Ecography, 34, 244–256. 10.1111/j.1600-0587.2010.06390.x

[ece34984-bib-0032] Odum, E. P. (1950). Bird populations of the Highlands (North Carolina) Plateau in relation to plant succession and avian invasion. Ecology, 31, 587–605. 10.2307/1931577

[ece34984-bib-0033] Oksanen, J. , Blanchet, G. , Friendly, M. , Kindt, R. , Legendre, P. , McGlinn, D. , … Wagner, H. (2017). vegan: Community ecology package. R package version 2.4‐4. Retrieved from https://cran.r-project.org/package=vegan

[ece34984-bib-0034] Orlóci, L. (1978). Multivariate analysis in vegetation research, 2nd ed. The Hague, The Netherlands: Dr. W. Junk B. V.

[ece34984-bib-0035] Pickett, S. T. A. , Collins, S. L. , & Armesto, J. J. (1987). Models, mechanisms and pathways of succession. The Botanical Review, 53, 335–371. 10.1007/BF02858321

[ece34984-bib-0036] Podani, J. , Ricotta, C. , & Schmera, D. (2013). A general framework for analyzing beta diversity, nestedness and related community‐level phenomena based on abundance data. Ecological Complexity, 15, 52–61. 10.1016/j.ecocom.2013.03.002

[ece34984-bib-0037] Podani, J. , & Schmera, D. (2011). A new conceptual and methodological framework for exploring and explaining pattern in presence‐absence data. Oikos, 120, 1625–1638. 10.1111/j.1600-0706.2011.19451.x

[ece34984-bib-0038] Ružička, M. (1958). Anwendung mathematisch‐statisticher Methoden in der Geobotanik (synthetische Bearbeitung von Aufnahmen). Biologia, Bratislava, 13, 647–661.

[ece34984-bib-0039] Schaefer, J. , Gido, K. , & Smith, M. (2005). A test for community change using a null model approach. Ecological Applications, 15, 1761–1771. 10.1890/04-1490

[ece34984-bib-0040] Shimadzu, H. , Dornelas, M. , & Magurran, A. E. (2015). Measuring temporal turnover in ecological communities. Methods in Ecology and Evolution, 6, 1384–1394. 10.1111/2041-210X.12438

[ece34984-bib-0041] van den Brink, P. J. , & ter Braak, C. J. F. (1999). Principal response curves: Analysis of time‐dependent multivariate responses of biological community to stress. Environmental Toxicology and Chemistry, 18, 138–148. 10.1002/etc.5620180207

[ece34984-bib-0042] Vellend, M. (2016). The theory of ecological communities. Princeton, NJ: Princeton University Press.

[ece34984-bib-0043] Warwick, R. M. , Clarke, K. R. , & Suharsono . (1990). A statistical analysis of coral community responses to the 1982–83 El Niño in the Thousand Islands, Indonesia. Coral Reefs, 8, 171–179.

[ece34984-bib-0044] Whittaker, R. H. (1972). Evolution and measurement of species diversity. Taxon, 21, 213–251. 10.2307/1218190

[ece34984-bib-0045] Winegardner, A. K. , Legendre, P. , Beisner, B. E. , & Gregory‐Eaves, I. (2017). Diatom diversity patterns over the past c. 150 years across the conterminous United States of America: Identifying mechanisms behind beta diversity. Global Ecology and Biogeography, 26, 1303–1315.

