# Supplementary material for: A temporal beta‐diversity index to identify sites that have changed in exceptional ways in space–time surveys
Source: Ecol Evol. 2019 Feb 18;9(6):3500–14. doi: 10.1002/ece3.4984 (PMC6434560; doi:10.1002/ece3.4984)
Supplement: Supplementary file 1 [file ECE3-9-3500-s001.pdf]

Supporting information to:

Legendre P. (2019) A temporal beta–diversity index to identify sites that have changed in exceptional ways in space–time surveys. *Ecology and Evolution* 2019; 00:1–15.

Appendix S1

SIMULATIONS INVOLVING ARTIFICIAL SURVEY DATA AT TIMES T1 AND T2

Introduction

This appendix reports the results of a simulation study carried out to determine if the new test of significance of the temporal beta change at individual sites (TBI) has correct type I error rates and is able to detect sites for which the response data had exceptionally high dissimilarities between time 1 (T1) and time 2 (T2). Simulations were done with random data generated in three different ways; six dissimilarity coefficients were used as TBI indices.

Data generation methods

Two methods were used to generate random community-like data in matrices **Mat.1** and **Mat.2**. The first one (gen.method=1) was to draw values at random from the Poisson distribution. The second method (gen.method=2) was to use random lognormal data. On the one hand, Poisson error regression is often recommended in GLM software to analyse count data such as species abundances. On the other hand, artificial data with lognormal distributions, rounded to integers, are often used to represent community composition data in simulation studies because their skewness is comparable to that often encountered in real community data (Preston 1948).

The random Poisson deviates were generated with a probability of occurrence (lambda parameter of the distribution) of 0.8. The generated data were skewed and contained approximately 45% zeros. For the lognormal data, the normal distribution generating the deviates that are exponentiated to produce random lognormal data had mean = 0 and standard deviation = 2.0. The values were rounded to the nearest integer. The generated data were highly skewed and contained approximately 36.5% zeros. Random lognormal data are much more highly skewed than random Poisson data.

It may be of interest to ecologists to identify sites that are exceptional in the changes to their community structure on the one hand, and sites that are exceptional in the changes to their environmental conditions on the other hand. So, in a third series of simulations, quantitative environmental data will be generated through random normal deviates; they will represent environmental variables in simulations. Real quantitative environmental data are often not normally distributed, but in many cases then can be normalized using data transformations. Qualitative variables (factors) will not be used in the simulations. How to handle them is described in section "Application to physical environmental or community trait data" of the main paper.

a. Dissimilarity coefficients

Six dissimilarity coefficients were used in the simulation study. Among the coefficients that are often used to analyse community composition data, we used the chord, Hellinger and log-chord distances, which belong to the Box-Cox family of distances (Legendre & Borcard 2018) and have the Euclidean property (a useful property for principal coordinate ordination, PCoA), and the percentage difference (Odum 1950) and Ružička (1958) dissimilarities, which are non-Euclidean dissimilarities and can

produce negative eigenvalues and complex axes in PCoA. The Euclidean distance was also included because it is the most widely used coefficient to analyse environmental data matrices transformed by standardization or ranging. Its behaviour for the analysis of community data will be assessed against the coefficients specialized for this type of data.

b. Permutation methods

The permutation method implemented in the TBI.R function and used in the simulations reported in the following sections of this Appendix is described in the main paper. Briefly, the data are permuted separately in each column, in the same way in matrices **Mat.1** (for T1) and **Mat.2** (for T2).

Permutation of entire rows of data is another method, widely used by ecologists in tests of significance in canonical analysis (RDA, CCA). That method was used in additional TBI simulations for the sake of the comparison; results of these additional simulations are summarized here. In the TBI test, entire rows were permuted in matrices **Mat.1** and **Mat.2** separately. The tests had correct type I error rates, so they were valid, but they had much lower power than the permutation method summarized in the previous paragraph. Hence permutation of entire rows of data should not be used to test the significance of TBI indices. That method is not included in the TBI.R function found in package *adespatial* on CRAN.

c. Simulations to estimate type I error rates

Type I error simulations will provide an assessment of the validity of the testing method. "A statistical testing procedure is valid if the probability of a type I error (rejecting H_0 when true) is no greater than α, the level of significance, for any α." (Edgington 1995, p. 37).

c.1. Simulation methods

c.1.1. *Simulation series 1*, community composition data, random Poisson deviates

Two subseries of simulations were carried out:

1.1. In the first subseries, the two matrices (**Mat.1** and **Mat.2**, of size $n \times p$) contained random Poisson deviates, as described in the introduction of this Appendix; Fig. S1.1. The permutation method was used with 999 random permutations. Simulation results are presented in Table S1.1.

1.2. In the second subseries of simulations, in addition to the random rata in mat.1a and mat.2a (which are called mat.1 and mat.2 in Fig. S1.1), a submatrix mat1.d containing *p3* new species, with frequencies zero, was added to mat.1a and a matrix mat2.d of the same size, containing random Poisson deviates, was added to mat.2a; see Fig. S1.2. Simulation results with *p3* = 6 are shown in Table S1.2.

The objective of the second subseries was to show the effect on the tests of significance of having extra species in the data matrices showing strong difference between T1 and T2 (species absent in **Mat.1** and present in **Mat.2**) but with only random differences among the sites. These extra species should have no effect on the results of TBI tests.

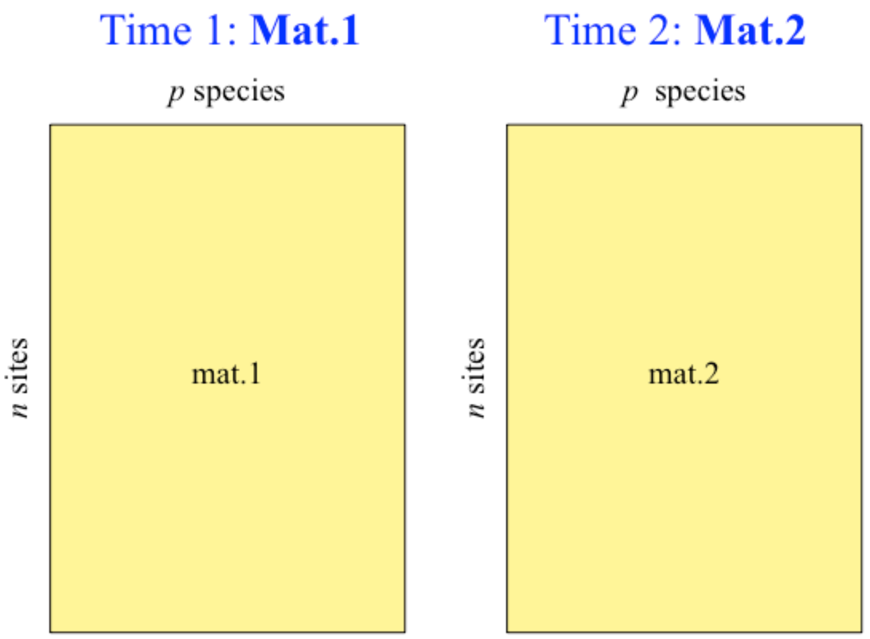

Fig. S1.1. Two data matrices (**Mat.1** and **Mat.2**) used in the type I error simulations. Sections mat.1 and mat.2 were filled with random numbers, so that H_0 was true. Note: **Mat.1** and **Mat.2** contain only one section each here; they will contain more sections in the next figures.

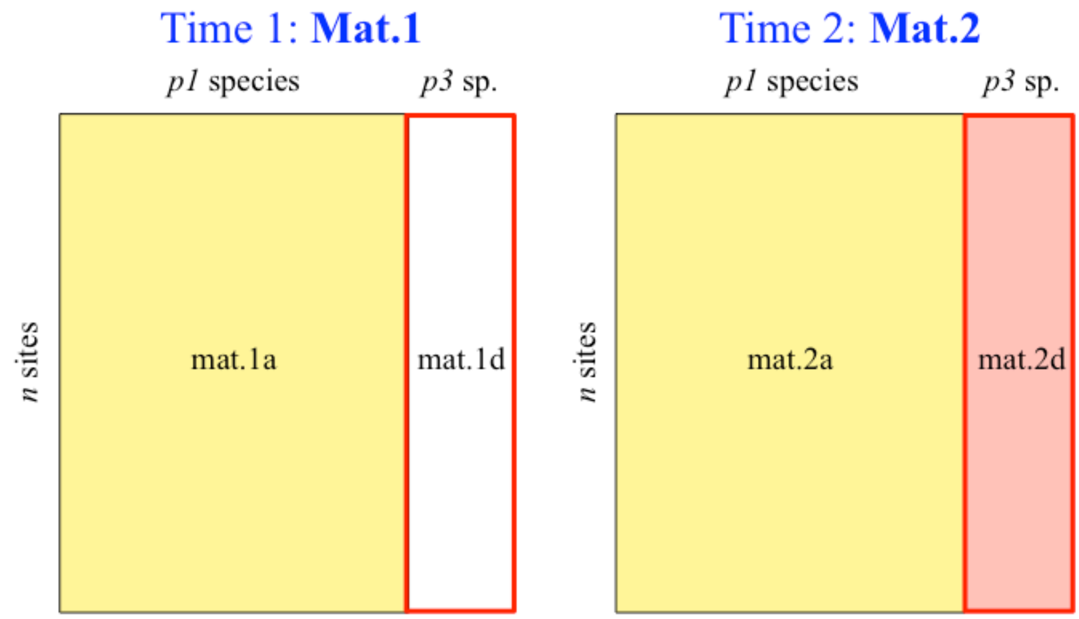

Fig. S1.2. Two data matrices (**Mat.1** and **Mat.2**) used in the type I error simulations; mat.1a and mat.2a contained random numbers, as in Fig. S1.1. In the simulations of subseries 2, mat.1d (white, containing zeros for *p3* new species) and mat.2d (pink, containing random numbers for the same number of species, *p3*) were joined to mat.1a and mat.2a.

c.1.2. *Simulation series 2*, community composition data, random lognormal deviates

Two subseries of simulations were carried out:

2.1. In the first subseries, the two matrices (mat.1 and mat.2, of size $n \times p$) contained random lognormal deviates, as described in the introduction of this Appendix; Fig. S1.1. The permutation method was used with 999 random permutations. Simulation results are presented in Table S1.3.

2.2. In the second subseries of simulations, in addition to the random rata in mat.1a and mat.2a (called mat.1 and mat.2 in Fig. S1.1), a submatrix mat1.d containing *p3* new species, with frequencies zero, was added to mat.1a and a matrix mat2.d of the same size, containing random lognormal deviates, was added to mat.2a; see Fig. S1.2. Simulation results with $p3 = 6$ are shown in Table S1.4.

The objective of the second subseries was the same as for subseries 1.2, using a different way of generating community composition-like data.

c.1.3. *Simulation series 3*, environmental data, random normal deviates

In this last series, quantitative environmental data were simulated using random normal deviates instead of species-like data. There were $n = 20$ sites and $p = 20$ variables in matrices **Mat.1** and **Mat.2**, as in Fig. S1.1. TBI tests were only computed with the Euclidean distance. The other distances investigated in the previous simulation series only make sense for community composition and other frequency-like data (Legendre & Legendre 2012, Chapter 7). The permutation method was used with 999 random permutations.

The data vectors were standardized as described in Appendix A2. Explanation: (a) the two data matrices are joined into a single data matrix, **Y** = rbind(**Mat.1**, **Mat.2**), before standardization. In this way, the differences in values of each variable for a given pair of sites in the two matrices will remain comparable to the differences of computed from the original unstandardized values; in this way, the distances computed between sites in T1 and T2 will be meaningful. This precaution is important when there are differences in means between T1 and T2. (b) Standardizing the variables insures that all variables will contribute the same variance to the calculation of the TBI indices; the variances will not depend on the physical units of the variables or other contingencies that make the variances unequal.

c.2. Results, type I error study

Results are presented in Tables S1.1 and S1.2 for random Poisson deviates, in Tables S1.3 and S1.4 for random lognormal deviates, and in Table S1.5 for normal deviates.

The simulations produced the expected result that type I error was always correct. The testing method is thus valid according to Edgington's (1995) definition shown above.

1. Examination of the tables of rejection rates of the null hypothesis (Table S1.1–S1.4) showed that the TBI tests had correct rates of type I error for the two community-like data generation methods (Poisson and lognormal) and all dissimilarity indices used, and this for all significance levels (α) considered, from $\alpha = 0.01$ to $\alpha = 0.50$. Deviations from the nominal significance levels, shown at the top of each table, were very small. Simulations involving random environmental-like quantitative data (Table S1.5) also showed correct levels of type I error with the Euclidean distance used in the computation of TBI indices.

2. The tables of rejection rates were divided into separate matrices per data generation and permutation methods, and transformed into squared differences (or squared errors) between the rejection rates and

the nominal significance levels α. The sum of the squared differences was computed for each matrix. Examination of the results (not shown in detail in this Appendix) showed no significant difference (Friedman's test) in type I error rejection rates between the data generation methods (Poisson or lognormal).

3. The additional species that were present in **Mat.2** but not in **Mat.1** (see Fig. S1.2) did not affect the type I error rates of the TBI tests. The rejection rates in Tables S1.1 and S1.2 (random Poisson deviates), and those in Tables S1.3 and S1.4 (random lognormal deviates), were very similar.

Note — The percentage difference and Ružička dissimilarity indices differ only by their denominators. The tests of significance of these two indices produce the same p-values if they are run with the same series of permuted vectors. Since the random number generator was started at the same value at the beginning of all simulation runs, it is normal that the rejection rates found for these two dissimilarities in TBI simulations be the same in the report tables (Tables S1.1 to S1.4).

d. Simulations to compare power of *D* indices

d.1. Simulation method

For the power simulations, some of the sites were generated with a strong difference between T1 and T2 whereas other sites only had random differences. The objective of these simulations was to determine if some dissimilarity functions were better suited to identify sites with strong differences in community composition data between T1 and T2 than other dissimilarity indices.

Data generation proceeded as follows for community composition data. *n1* sites were assigned to a first group that differed in composition between T1 and T2, whereas *n2* sites only had random variation between T1 and T2.

1. The *n1* sites received strong differences between T1 and T2, as follows (Fig. S1.3):

• A first group of *p1* species received random abundance values in mat.1a.

• A second group of *p2* species received random abundance values in mat.2b.

• In **Mat.1**, the *p2* species received the values of mat.2b times a contribution constant (parameter *contr*) with values between 0 and 1. With *contr* = 0, the differences between **Mat.1** and **Mat.2** for the first 5 sites were so strong that H_0 was rejected with very high rates and the first 5 sites were nearly always identified as exceptional, except when the Euclidean *D* was used as TBI index. The value *contr* = 0 was too strong to produce useful simulation results. Hence the value *contr* = 0.2 was used in the power simulations with random Poisson deviates reported below. For the power simulations with random lognormal deviates, *contr* was either 0.01 or 0.02, as specified below. A higher value of *contr* reduces the test power.

• In **Mat.2**, the *p1* species received the values of mat.1a times the same contribution constant (*contr*) as in the previous paragraph.

The generated abundance-like values were either random Poisson or random lognormal deviates, as described at the beginning (section "Data generation methods") of this Appendix.

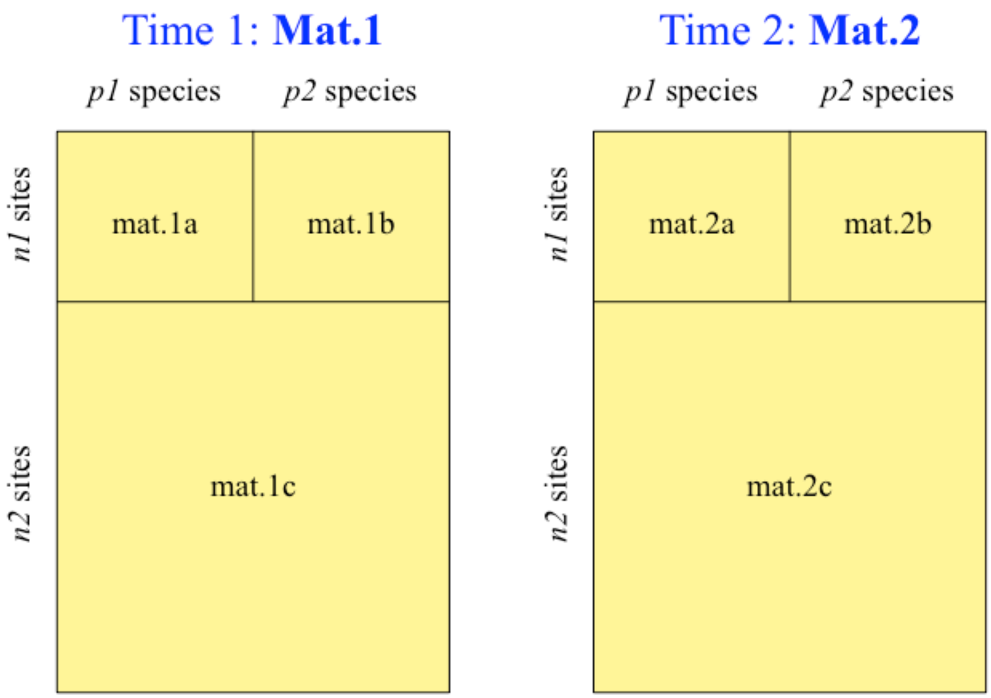

Fig. S1.3. Two matrices (**Mat.1** and **Mat.2**) used in the power simulations. Abundances in mat.1a and mat.2b were generated independently using either random Poisson or random lognormal deviates. Submatrix mat.1b received a fraction of the abundances in mat.2b and mat.2a received a contribution of the abundances in mat.1a. The values of these contributions are described in the text and depend of the random data generator used. Submatrices mat.1c and mat.2c received random deviates drawn from the same statistical population.

2. The *n2* sites received random abundance-like values, either Poisson or lognormal. Hence the differences between T1 and T2 were random for these sites (Fig. S1.3).

In the results reported below, $n1 = 5$, $n2 = 15$, $p1 = p2 = 10$. 1000 data sets were independently generated and analysed for each reported rejection rate (0.01, 0.05, 0.10) in Tables S1.6 and S1.7 reporting the results.

d.1.1. Power differences among D indices, community composition data

d.1.1.1. *Simulations using random Poisson deviates*

Two subseries of simulations were carried out:

1. In the first subseries, all matrices contained random Poisson deviates, structured following Fig. S1.3.

2. In addition, a submatrix mat1.d containing *p3* new species, with frequencies zero, was added to **Mat.1** and a matrix mat2.d of the same size, containing random Poisson deviates, was added to **Mat.2**, as in the subseries 2 simulations for type I error. See Fig. S1.4.

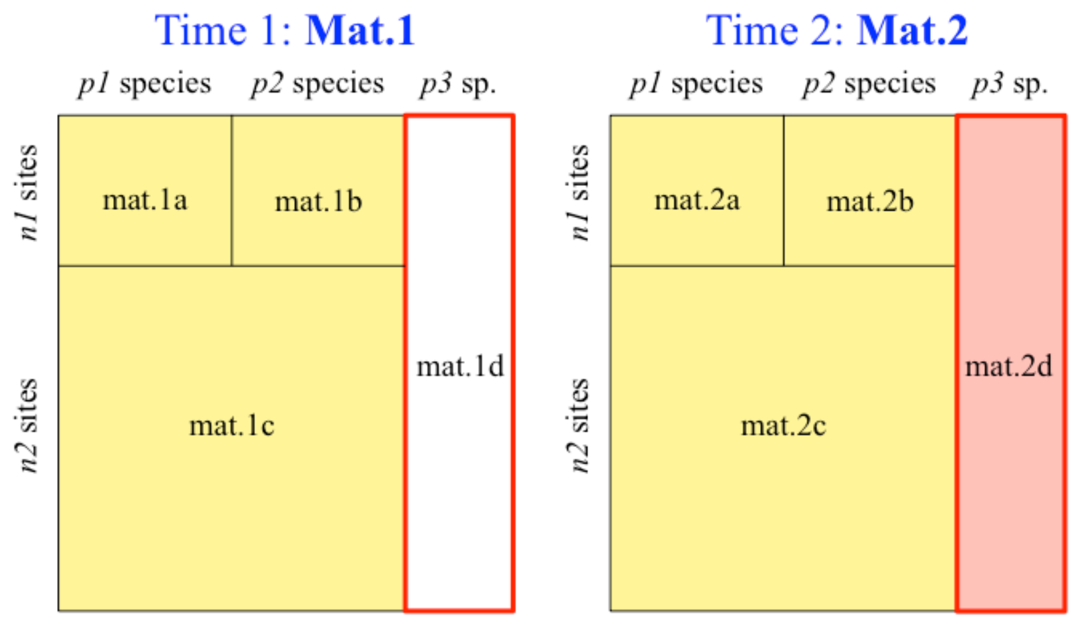

Fig. S1.4. Two matrices (**Mat.1** and **Mat.2**) used in additional power simulations with random deviates. In these simulations, mat.1d (white, containing zeros for *p3* new species) and mat.2d (pink, containing random lognormal or Poisson deviates for the same number of species, *p3*) were joined to mat.1a-b-c and mat.2a-b-c. H_0 was true for the *p3* species found in mat.1d and mat.2d.

The objective of the second subseries was to show the effect on the tests of significance of having extra species in the data matrices showing strong difference between T1 and T2 (species absent in **Mat.1** and present in **Mat.2**) but with only random differences among the sites. These extra species should have little effect, if any, on the TBI tests of significance.

d.1.1.2. *Simulations using random lognormal deviates*

Two subseries of simulations were carried out with random lognormal deviates. Data were structured following Fig. S1.3.

1. In the first subseries, the contributions of the mat.2b data to the mat.1b data, and of the mat.1a data to the mat.2a data, was determined by *contr* = 0.01.

2. In the second subseries, the contribution parameter (*contr*) had the value 0.02.

d.1.2. Power with simulated environmental data

For simulation involving data simulating pseudo-environmental variables, the construction of the data matrices followed the same method as in simulation subseries 1 above (*n1* = 5, *n2* = 15, *p1* = *p2* = 10), except that the data were random normal deviates standardized as described in Appendix A2.

d.1.3. Differences in power for different values of *n1* and *n2*

Power to detect an effect in statistical tests is well-known to be a function of three parameters: the importance of the effect to be detected, the significance level α, and the number of observations *n*. Additional simulations were conducted to detect the effect on power of the number of exceptional sites

(*n1*) and the number of sites with random variation (*n2*) in the study. Community composition was simulated using random lognormal deviates.

d.2. Results, power study

Results are presented in Tables S1.6 and S1.7 for simulated community composition data and in Table S1.8 for simulated environmental data.

d.2.1. Power differences among D indices, community composition data

Power is the ability to detect an effect when one is present in the data. In the simulations reported here, we know that rather large effects were present in all data sets because the simulation function had generated it in the data.

From the simulation results for community composition data (simulation series 1: Table S1.6, Fig. S1.5; simulation series 2: Table S1.7, Fig. S1.6), we can make the following observations, working from the bottom of the graphs up:

1. All simulations involving the Euclidean distance for the computation of TBI indices had dismally low power (triangles pointing down). TBI indices computed with the Euclidean distance hardly ever detected the presence of exceptional sites in the species-like data files simulated with Poisson or lognormal deviates. The Euclidean distance should not be used for TBI tests of community composition data.

2. For data generated with Poisson or lognormal distributions, the most powerful TBI tests were computed with the percentage difference (aka Bray-Curtis) and Ružička dissimilarities, followed by the group {Hellinger, log-chord} distances which produced very similar results with species-like data simulated with Poisson random deviates, and log-chord distances for community data simulated with lognormal deviates. TBI tests based on the chord dissimilarity had the lowest power among the distances that produced usable tests. The log-chord distance was expected to be the most appropriate (and thus produce more powerful tests that the chord or Hellinger distances) with lognormally distributed data since the log transformation, which is the first transformation in the calculation of that distance, makes the random data normal before the chord transformation is computed.

3. When sites had entirely different species compositions between T1 and T2, the TBI test had maximum power: it always rejected H_0 at significance levels of 0.05 and 0.10, and in 98.7% of the cases at level 0.01. In temporal studies, this situation may arise when a site has been subjected to an important environmental change between T1 and T2. The change may be natural or man-made, like a lake environment going from a prairie to agricultural or urban. The situation was simulated by setting the contribution parameter to the value *contr*=0 for the generation of data in submatrices mat1b and mat2b (see Fig. S1.3). Similar results were obtained with all dissimilarity indices tested: percentage difference, Ruzicka, chord, Hellinger and log-chord distances.

The best combination for TBI tests of community composition data with maximum power is to use the percentage difference or the Ružička indices. These two dissimilarities can also be decomposed into species losses (*B*/*den*) and gains (*C*/*den*), which can be used to examine the processes of losses and gains at the site level and to produce B-C plots.

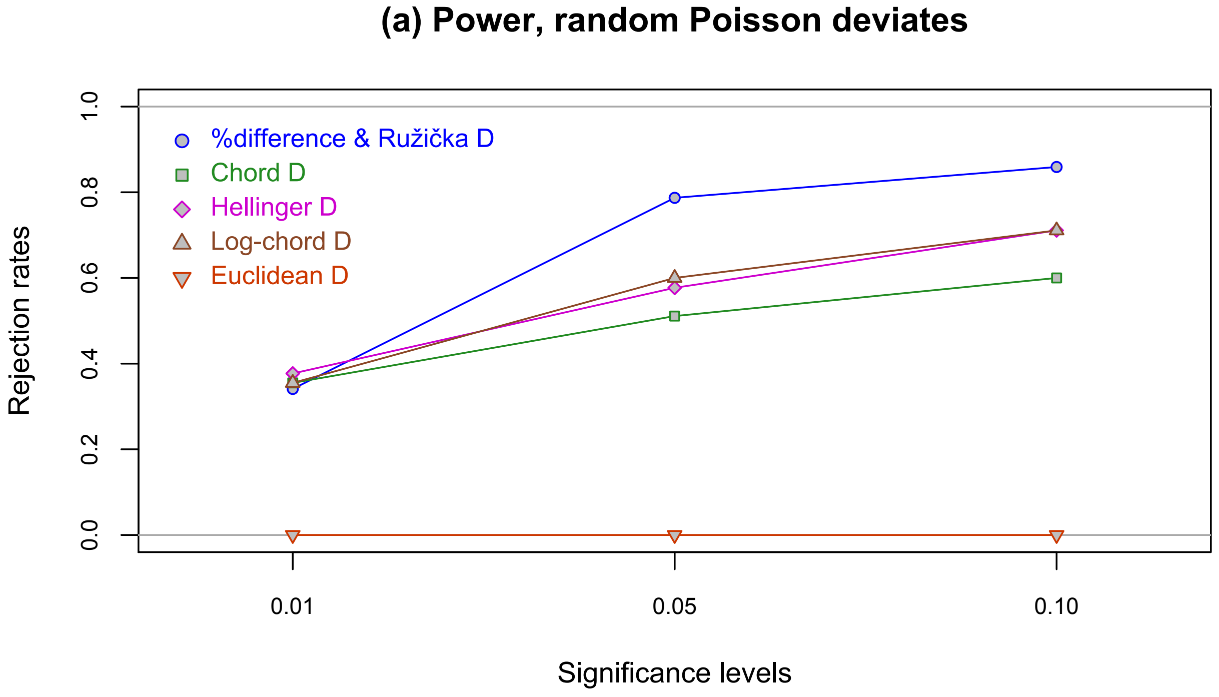

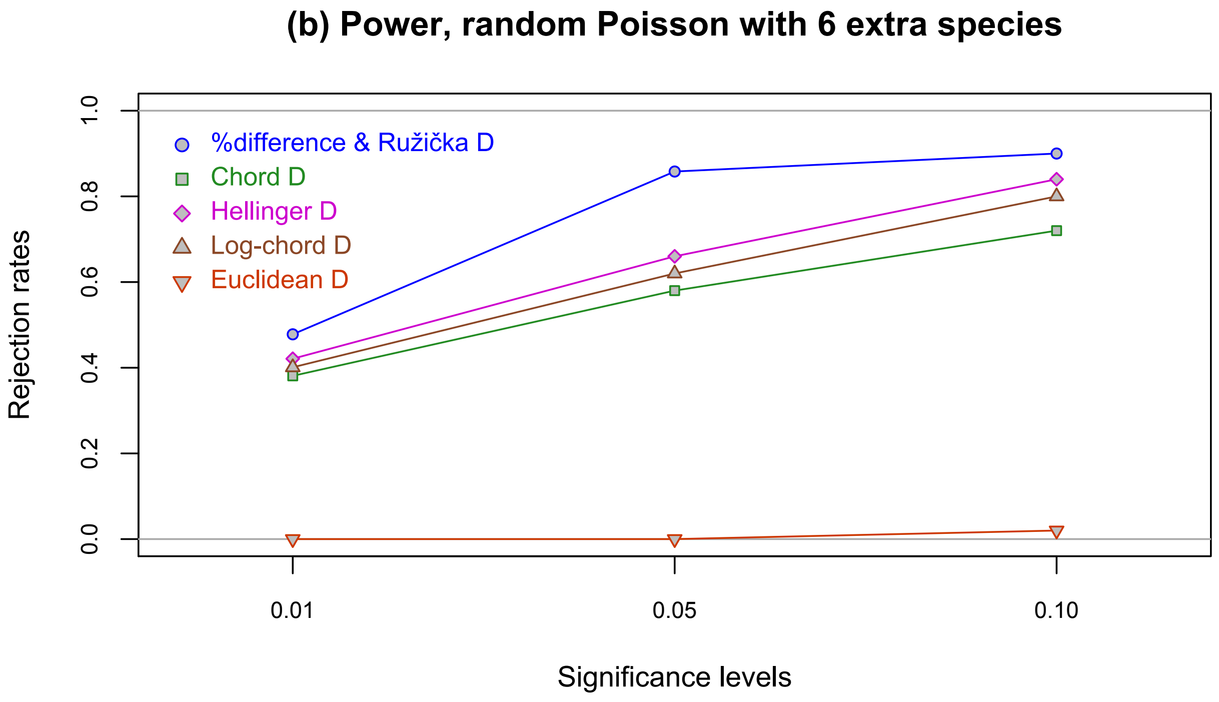

Fig. S1.5. Power study, random Poisson deviates. Rates of rejection of H_0 through the simulations obtained with five dissimilarity coefficients, **(a)** without (Fig. S1.3) and **(b)** with extra species (Fig. S1.4). Rejection rates are reported for three significance levels alpha: 0.01, 0.05 and 0.10 (abscissa).

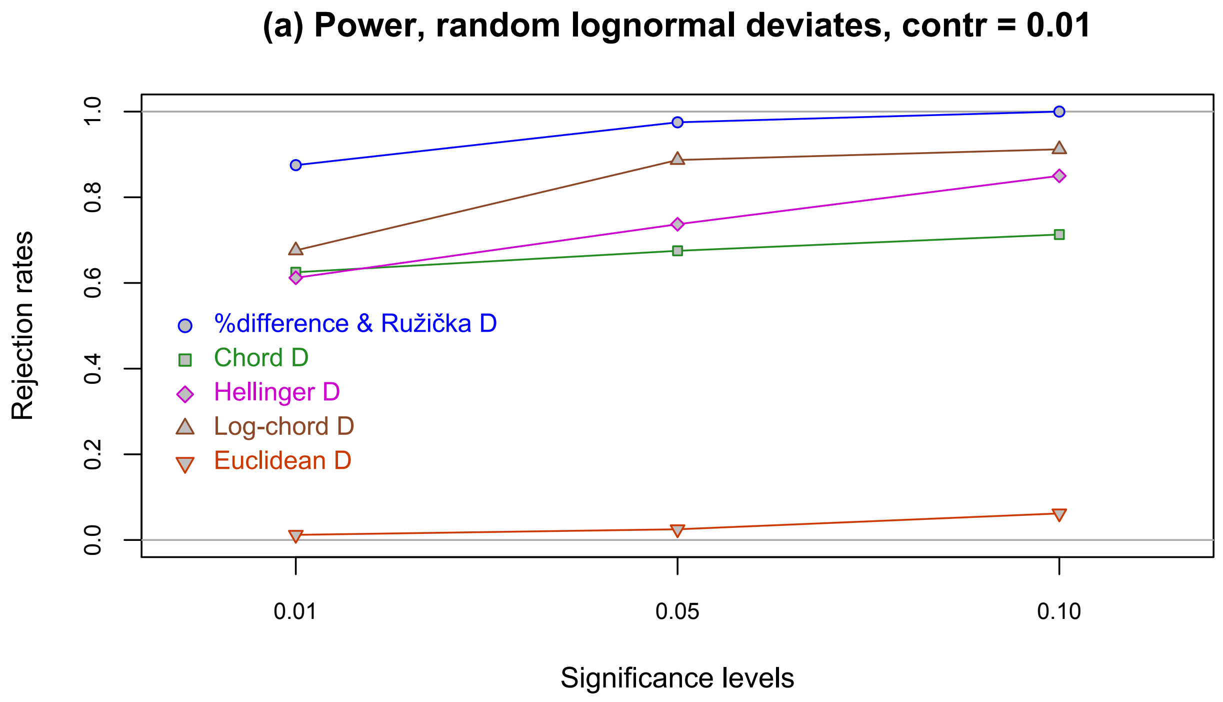

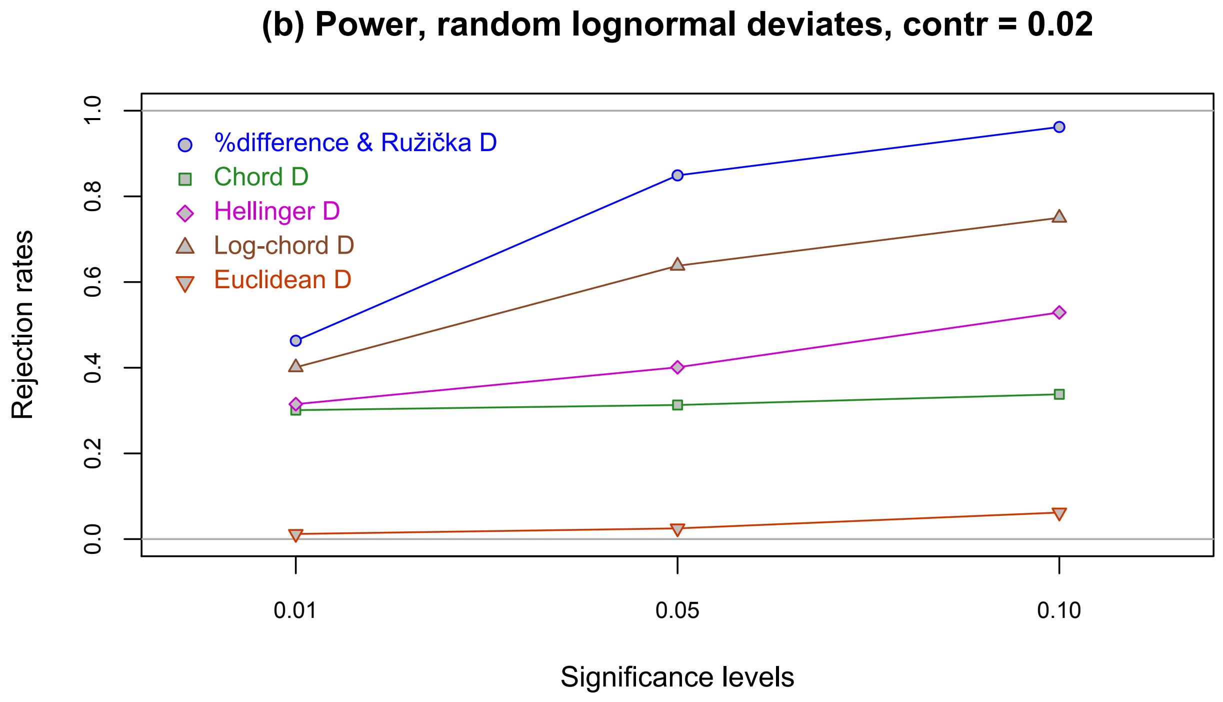

Fig. S1.6. Power study, random lognormal deviates. Rates of rejection of H_0 through the simulations obtained with five dissimilarity coefficients, using two values of the contribution parameter: **(a)** *contr* = 0.01, **(b)** *contr* = 0.02. Rates of rejection of H_0 are reported for three significance levels alpha: 0.01, 0.05 and 0.10 (abscissa).

d.2.2. Power with simulated environmental data

In the simulations representing environmental variables with simulated standardized random normal deviates, there were $n1$ = 5 and $n2$ = 15 sites, $p1 = p2$ = 10 variables. The contribution parameter was *contr* = 0.05. The only distance tested in the simulation study was the Euclidean distance. 1000 data sets were independently generated and analysed for each reported rejection rate (0.01, 0.05, 0.10). The rates of rejection of H_0 are presented in Table S1.8. Power was high enough to recommend the test for analysis of standardized environmental data.

d.2.3. Differences in power for different values of *n1* and *n2*

The simulations conducted to detect the effect on power of the number of exceptional sites ($n1$) and the number of sites with random variation ($n2$) in the study produced the following results.

1. The number of sites n was 20 in all simulations, with the number of affected sites $n1$ = varying from 1 to 19; $n2 = (n - n1)$. The simulations used the percentage difference index and *contr* = 0.02, 1000 independent simulations and 999 permutations per test.

The results show that for tests of significance at level alpha = 0.05, optimal power was obtained with $n1$ = 1 to 9 in simulations (Fig. S1.7a). The test can be recommended for data sets with $n1$ smaller than $n/2$. Tests carried out with $n1$ equal to or larger than $n/2$ can still be used but they have lower power.

2. For $n1 = n/4$, simulations were repeated for different values, with $n = \{8,16,24,32,40,48,56,64\}$ and $n1 = \{2,4,6,8,10,12,14,16\}$. Again, the simulations used random lognormal data, the percentage difference index, *contr* = 0.02, 1000 independent simulations and 999 permutations per test.

Rejection rates are reported in Fig. S1.7b for three significance levels alpha: 0.01, 0.05 and 0.10. Power remained constant over all values of n investigated.

d.2.4. Summary of the power study

Power of the test was high when the effect was strong, and as long as the proportion of sites with an effect was smaller than $n/2$ (Fig. S1.7a). For a fixed proportion of affected sites, power did not increase when the total number of sites n in the study was larger (Fig. S1.7b).

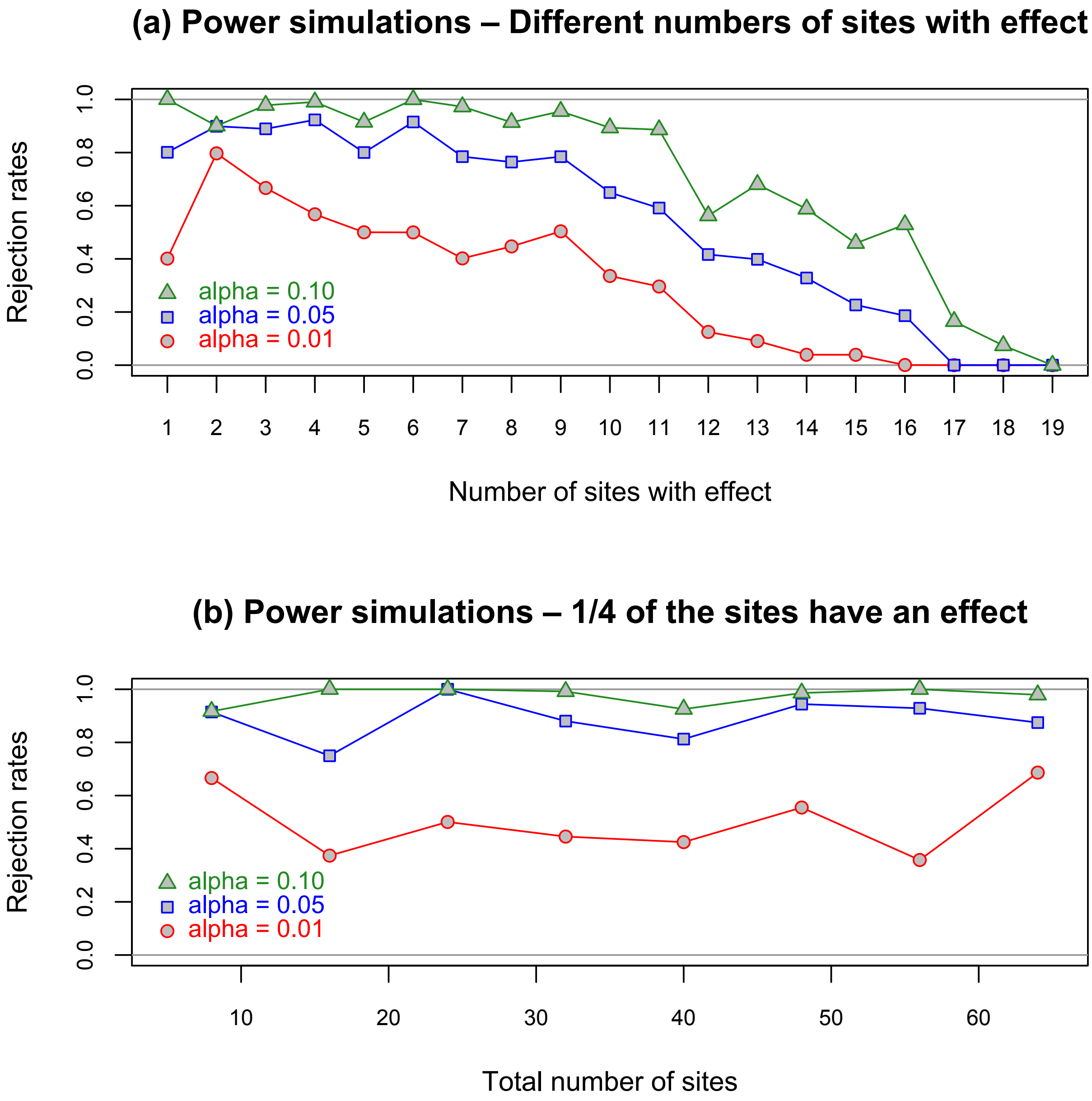

Fig. S1.7. Rejection rates of the TBI tests in power simulations. Rates of rejection of H_0 through the simulations are reported for three significance levels alpha: 0.01, 0.05 and 0.10. **(a)** Different numbers of sites received an effect (abscissa, *n1* = 1 to 19), i.e. a difference in community composition between T1 and T2. There were 20 sites in total in each simulation. **(b)** The proportion of sites with an effect was kept constant, here *n1* = *n*/4, for different values of *n* (abscissa). The rejection rate is the mean proportion of the sites with an effect that were identified as significant at the stated alpha level.

References

Edgington, E. S. (1995) *Randomization Tests, 3rd edn.* Marcel Dekker, New York.

Legendre, P. & Borcard, D. (2018) Box-Cox-chord transformations for community composition data prior to beta diversity analysis. *Ecography* **41**, 1–5.

Legendre, P. & Legendre, L. (2012) *Numerical ecology, 3rd English edn*. Elsevier Science BV, Amsterdam.

Odum, E. P. (1950) Bird populations of the Highlands (North Carolina) Plateau in relation to plant succession and avian invasion. *Ecology* **31**, 587–605.

Preston, F. W. (1948) The commonness, and rarity, of species. *Ecology* **29**, 254–283.

Ružička, M. (1958) Anwendung mathematisch-statisticher Methoden in der Geobotanik (synthetische Bearbeitung von Aufnahmen). *Biologia, Bratislava* **13**, 647–661.

Table S1.1. Type I error rates of the test of the TBI *D* indices shown in the first column: rejection rates (i.e. number of rejections of H_0 divided by the number of simulations, which was 1000) of the TBI test when there were no exceptional sites in the simulated data. The data were drawn from a random Poisson distribution; n = 20 sites, p = 20 species (Fig. S1.1). All tests involved 999 random permutations. Simulation series 1: data at all sites and both times came from the same statistical population, hence H_0 was true.

	Nominal significance levels						
	0.01	0.05	0.10	0.20	0.30	0.40	0.50
% difference	0.000	0.034	0.108	0.208	0.275	0.335	0.491
Ružička	0.000	0.033	0.108	0.208	0.275	0.333	0.492
Chord	0.000	0.042	0.075	0.200	0.292	0.383	0.475
Hellinger	0.000	0.042	0.100	0.175	0.300	0.367	0.517
Log-chord	0.000	0.050	0.117	0.183	0.300	0.375	0.483
Euclidean	0.000	0.025	0.094	0.200	0.319	0.400	0.494

Table S1.2. Type I error rates of the test of the TBI *D* indices shown in the first column. See caption of Table S1.1. The data were drawn from a random Poisson distribution; n = 20 sites. Simulation series 2: for the basic $p1$ = 20 species, data at all sites and both times came from the same statistical population. In addition, T2 had $p3$ = 6 species more than T1 (Fig. S1.2). All tests involved 999 random permutations. For these 6 species, there were no differences among the sites besides random variation; hence H_0 was still true.

	Nominal significance levels						
	0.01	0.05	0.10	0.20	0.30	0.40	0.50
% difference	0.017	0.042	0.092	0.183	0.267	0.425	0.541
Ružička	0.017	0.042	0.091	0.183	0.267	0.425	0.541
Chord	0.000	0.062	0.112	0.212	0.312	0.450	0.525
Hellinger	0.000	0.075	0.112	0.213	0.312	0.375	0.550
Log-chord	0.000	0.075	0.100	0.238	0.312	0.400	0.550
Euclidean	0.008	0.050	0.100	0.208	0.300	0.383	0.508

Table S1.3. Type I error rates of the test of the TBI *D* indices shown in the first column: rejection rates (i.e. number of rejections of H_0 divided by the number of simulations, which was 1000) of the TBI test when there were no exceptional sites in the simulated data. The data were drawn from a random lognormal distribution; n = 20 sites, p = 20 species (Fig. S1.1). All tests involved 999 random permutations. Simulation series 1: data at all sites and both times came from the same statistical population, hence H_0 was true.

	Nominal significance levels						
	0.01	0.05	0.10	0.20	0.30	0.40	0.50
%difference *D*	0.014	0.064	0.121	0.193	0.293	0.386	0.486
Ružička *D*	0.014	0.064	0.121	0.193	0.293	0.386	0.486
Chord *D*	0.000	0.038	0.100	0.188	0.251	0.388	0.513
Hellinger *D*	0.000	0.050	0.100	0.175	0.263	0.375	0.562
Log-chord *D*	0.000	0.025	0.076	0.213	0.313	0.425	0.512
Euclidean *D*	0.000	0.050	0.112	0.188	0.312	0.413	0.500

Table S1.4. Type I error rates of the test of the TBI *D* indices shown in the first column. See caption of Table S1.3. The data were drawn from a random lognormal distribution; n = 20 sites. All tests involved 999 random permutations. Simulation series 2: for the basic $p1$ = 20 species, data at all sites and both times came from the same statistical population. In addition, T2 had $p3$ = 6 species more than T1 (Fig. S1.2). For these 6 species, there were no differences among the sites besides random variation; hence H_0 was still true.

	Nominal significance levels						
	0.01	0.05	0.10	0.20	0.30	0.40	0.50
%difference *D*	0.022	0.061	0.105	0.210	0.294	0.389	0.500
Ružička *D*	0.022	0.061	0.105	0.210	0.294	0.389	0.500
Chord *D*	0.017	0.061	0.100	0.222	0.300	0.411	0.517
Hellinger *D*	0.017	0.055	0.111	0.194	0.273	0.368	0.501
Log-chord *D*	0.022	0.072	0.116	0.194	0.273	0.401	0.506
Euclidean *D*	0.017	0.056	0.133	0.194	0.322	0.405	0.505

Table S1.5. Type I error rates of the test of TBI indices computed using the Euclidean distance. Simulation series 3: the data were drawn from a random normal distribution; n = 20 sites, p = 20 variables. All tests involved 999 random permutations. Data at all sites and both times came from the same statistical population, hence H_0 was true.

	Nominal significance levels						
	0.01	0.05	0.10	0.20	0.30	0.40	0.50
Euclidean *D*	0.007	0.050	0.100	0.193	0.321	0.421	0.528

Table S1.6. Power analysis of TBI *D* indices shown in the first column, random Poisson deviates. There were $n1$ = 5 exceptional sites in the simulated data. The results in the table are the mean rejection rates of the TBI test for these 5 sites, computed as the number of rejections of H_0 divided by the number of simulations, which was 1000. (A) Data with $p1$ and $p2$ species only (Fig. S1.3). (B) $p3$ = 6 extra species with values of 0 in Mat.1 and random Poisson deviates in all sites of Mat.2 (Fig. S1.4); H_0 is true for these $p3$ species.

		(A) p1 and p2 species			(B) p1, p2 and p3 species		
		Significance levels			Significance levels		
		0.01	0.05	0.10	0.01	0.05	0.10
%difference *D*	1	0.341	0.787	0.859	0.478	0.858	0.900
Ružička *D*	1	0.341	0.787	0.859	0.478	0.858	0.900
Chord *D*	1	0.355	0.511	0.600	0.381	0.580	0.720
Hellinger *D*	1	0.377	0.577	0.711	0.421	0.660	0.840
Log-chord *D*	1	0.355	0.600	0.711	0.401	0.620	0.800
Euclidean *D*	1	0.000	0.000	0.000	0.000	0.000	0.020

Table S1.7. Power analysis of TBI *D* indices shown in the first column, random lognormal deviates. There were 5 exceptional sites in the simulated data. The results in the table are the mean rejection rates of the TBI test for these 5 sites, computed as the number of rejections of H_0 divided by the number of simulations, which was 1000. Data with *p1* and *p2* species only (Fig. S1.3). There were no extra species in these simulations ($p3 = 0$). The contribution parameter of these simulations varied: (A) *contr* = 0.01, (B) *contr* = 0.02.

		(A) *contr* = 0.01			(B) *contr* = 0.02		
		Significance levels			Significance levels		
		0.01	0.05	0.10	0.01	0.05	0.10
%difference *D*	1	0.875	0.975	1.000	0.463	0.849	0.962
Ružička *D*	1	0.875	0.975	1.000	0.463	0.849	0.962
Chord *D*	1	0.625	0.675	0.713	0.301	0.313	0.338
Hellinger *D*	1	0.612	0.737	0.850	0.315	0.401	0.529
Log-chord *D*	1	0.676	0.887	0.912	0.401	0.638	0.750
Euclidean *D*	1	0.012	0.025	0.062	0.012	0.025	0.062

Table S1.8. Power analysis of TBI indices computed using the Euclidean distance, random normal deviates. There were 5 exceptional sites in the simulated data. The results in the table are the mean rejection rates of the TBI test for these 5 sites, computed as the number of rejections of H_0 divided by the number of simulations, which was 1000. Data with p1 and p2 species only. There were no extra variables in these simulations ($p3 = 0$); *contr* = 0.05.

	contr = 0.05		
	Significance levels		
	0.01	0.05	0.10
Euclidean *D*	0.627	0.772	0.843
