# Supplementary material for: A temporal beta‐diversity index to identify sites that have changed in exceptional ways in space–time surveys
Source: Ecol Evol. 2019 Feb 18;9(6):3500–14. doi: 10.1002/ece3.4984 (PMC6434560; doi:10.1002/ece3.4984)
Supplement: Supplementary file 2 [file ECE3-9-3500-s002.pdf]

Appendix S2

AN R FUNCTION TO STANDARDIZE ENVIRONMENTAL DATA PRIOR TO TBI ANALYSIS

```
#' Special standardization for environmental data prior to TBI analysis.
#'
#' After standardization, all variables will have the same weight (i.e. they will
#' all contribute the same variance) in the calculation of TBI indices.
#'
#' @param mat1 First data matrix, class matrix or data.frame.
#' @param mat2 Second data matrix, class matrix or data.frame.
#' @param non.neg=TRUE : make the data non-negative before scaling (recommended).
#'        non.neg=FALSE: keep standardized data with signs (due to centring).
#'
#' @return A list with the two matrices standardized as described above.
#'
#' @details
#' The two data sets are joined into a single data matrix, Y = rbind(Y.T1, Y.T2).
#' Y is standardized [Y.stand = scale(Y)], then it is separated into two matrices
#' of the sizes of the original data matrices before analysis with function TBI().
#'
#' Explanation:
#' (a) the two data matrices are joined into a single data matrix, Y = rbind(Y.T1,
#' Y.T2), before standardization. In this way, the differences in values of each
#' variable for a given pair of sites in the two tables will remain comparable
#' to the differences computed from the original unstandardized values; in this
#' way, the distances computed between sites in T1 and T2 will be meaningful.
#' Important when there are differences in means and variances between T1 and T2.
#' (b) Standardizing the variables insures that all variables will contribute the
#' same variance to the calculation of the TBI indices; the variances will not
#' depend on the physical units of the variables or other contingencies that make
#' the variances unequal.
#'
#' Argument non.neg=TRUE makes all values ≥ 0. Subtracting the matrix overall
#′ minimum from all values does not change the Euclidean distances among the sites.
#'
#' @author  Pierre Legendre \email{pierre.legendre@@umontreal.ca}, 2018
'scale.for.TBI' <-
      function(mat1,mat2,
                   non.neg=TRUE)
      {
      mat1 <- as.matrix(mat1)
      mat2 <- as.matrix(mat2)
      dim.1 <- dim(mat1)
      dim.2 <- dim(mat2)
      if(!is.numeric(mat1)) stop("First data matrix not numeric")
      if(!is.numeric(mat2)) stop("Second data matrix not numeric")
      if(dim.1[1] != dim.2[1]) stop("Data sets have different numbers of rows")
      if(dim.1[2] != dim.2[2]) stop("Data sets have different numbers of columns")
      n12 <- dim.1[1]
      #
      tmp <- scale(rbind(mat1,mat2))
      if(non.neg) tmp <- tmp - min(tmp)
      mat1 <- tmp[1:n12,]
      mat2 <- tmp[(n12+1):(2*n12),]
      list(mat1=mat1, mat2=mat2)
      }
```
