# Supplementary material for: A temporal beta‐diversity index to identify sites that have changed in exceptional ways in space–time surveys
Source: Ecol Evol. 2019 Feb 18;9(6):3500–14. doi: 10.1002/ece3.4984 (PMC6434560; doi:10.1002/ece3.4984)
Supplement: Supplementary file 3 [file ECE3-9-3500-s003.pdf]

Appendix S3

RESULTS OF CALCULATIONS WITH R FUNCTION TBI(), INSECTICIDE EXPERIMENT

Pyrifos insect treatment data: compare survey #4 (one week after the insecticide treatment) to survey #11 (after full recovered from treatment). # indicate comments added to the output files.

```
library(vegan)
data(pyrifos)
survey4.order = c(38,39,41,47,37,44,40,46,43,48,42,45)
survey11.order = c(122,123,125,131,121,128,124,130,127,132,126,129)
```

1. Comparison based upon species abundance data, percentage difference D

```
( res1 <- TBI(pyrifos[survey4.order,], pyrifos[survey11.order,], method="%diff",
nperm=9999, BCD=TRUE, test.t.perm=TRUE, clock=TRUE) )
# Computation time = 47.247000  sec

-----
$TBI
 [1] 0.4332125 0.4490831 0.4048151 0.4593321 0.4958159 0.4392330 0.4884889 0.4851041
 [9] 0.4740264 0.6205484 0.7345825 0.6721440

$p.TBI
 [1] 0.8749 0.8082 0.9582 0.7518 0.5173 0.8588 0.5635 0.5938 0.6584 0.0192 0.0001 0.0018


$p.adj
 [1] 1.0000 1.0000 1.0000 1.0000 1.0000 1.0000 1.0000 1.0000 1.0000 0.1920 0.0012 0.0198

$BCD.mat
        B/(2A+B+C) C/(2A+B+C) D=(B+C)/(2A+B+C) Change
Site.1   0.1616465  0.2715660        0.4332125    +    # Untreated
Site.2   0.1973186  0.2517645        0.4490831    +    # Untreated
Site.3   0.2305092  0.1743059        0.4048151    -    # Untreated
Site.4   0.2643243  0.1950077        0.4593321    -    # Untreated
Site.5   0.2303800  0.2654359        0.4958159    +    # Treated, 0.1 microgram/L
Site.6   0.1980843  0.2411487        0.4392330    +    # Treated, 0.1 microgram/L
Site.7   0.2425404  0.2459484        0.4884889    +    # Treated, 0.9 microgram/L
Site.8   0.1854199  0.2996843        0.4851041    +    # Treated, 0.9 microgram/L
Site.9   0.1901665  0.2838599        0.4740264    +    # Treated, 6 micrograms/L
Site.10  0.3094316  0.3111168        0.6205484    +    # Treated, 6 micrograms/L
Site.11  0.3232546  0.4113279        0.7345825    +    # Treated, 44 micrograms/L
Site.12  0.1829121  0.4892319        0.6721440    +    # Treated, 44 micrograms/L

$BCD.summary   # Here the BCD summary is computed for the 12 mesocosms, not the 8 treated
 mean(B/den) mean(C/den)   mean(D)  B/(B+C)  C/(B+C) Change
   0.2263323   0.2866998 0.5130322 0.441166 0.558834    +

$t.test_B.C    # Here the tests is computed for the 12 mesocosms, not for the 8 treated
               mean(C-B)       Stat    p.param p.perm    p<=0.05
Paired t.test  0.06036748 -2.132286 0.05635548 0.0383          # Permutation test signif.

$BC
[1] NA

-----
```

```
# Additional result -
# Paired t-test comparing the B and C stat. ($BCD.mat above) for the 8 treated mesocosms

$t.test_B.C
               mean(C-B)      Stat    p.param p.perm   p<=0.05
Paired t.test  0.08569554 -2.463362 0.04325165 0.0066       *  # Both tests significant
```

---

2. Comparison based upon species occurrence (i.e. presence-absence) data, Sørensen D

```
( res2 <- TBI(pyrifos[survey4.order,], pyrifos[survey11.order,], method="sorensen",
nperm=9999, BCD=TRUE, test.t.perm=TRUE, clock=TRUE) )
# Computation time = 32.808000  sec

-----
$TBI
 [1] 0.4390244 0.4324324 0.4457831 0.4705882 0.4666667 0.4358974 0.5000000 0.4153846
 [9] 0.4545455 0.6800000 0.7551020 0.6595745

$p.TBI
 [1] 0.8281 0.8660 0.8051 0.6656 0.6858 0.8487 0.4960 0.9181 0.7671 0.0016 0.0001 0.0042

$p.adj
 [1] 1.0000 1.0000 1.0000 1.0000 1.0000 1.0000 1.0000 1.0000 1.0000 0.0176 0.0012 0.0420

$BCD.mat
        B/(2A+B+C) C/(2A+B+C) D=(B+C)/(2A+B+C) Change
Site.1   0.1463415  0.2926829        0.4390244    +   # Untreated
Site.2   0.1891892  0.2432432        0.4324324    +   # Untreated
Site.3   0.2048193  0.2409639        0.4457831    +   # Untreated
Site.4   0.2205882  0.2500000        0.4705882    +   # Untreated
Site.5   0.1733333  0.2933333        0.4666667    +   # Treated, 0.1 microgram/L
Site.6   0.1666667  0.2692308        0.4358974    +   # Treated, 0.1 microgram/L
Site.7   0.2105263  0.2894737        0.5000000    +   # Treated, 0.9 microgram/L
Site.8   0.1384615  0.2769231        0.4153846    +   # Treated, 0.9 microgram/L
Site.9   0.1363636  0.3181818        0.4545455    +   # Treated, 6 micrograms/L
Site.10  0.2800000  0.4000000        0.6800000    +   # Treated, 6 micrograms/L
Site.11  0.2857143  0.4693878        0.7551020    +   # Treated, 44 micrograms/L
Site.12  0.1276596  0.5319149        0.6595745    +   # Treated, 44 micrograms/L

$BCD.summary   # Here the BCD summary is computed for the 12 mesocosms, not the 8 treated
 mean(B/den) mean(C/den)   mean(D)   B/(B+C)   C/(B+C) Change
    0.189972   0.3229446 0.5129166 0.3703759 0.6296241    +

$t.test_B.C    # Here the test is computed for the 12 mesocosms, not for the 8 treated
               mean(C-B)      Stat      p.param p.perm   p<=0.05
Paired t.test   0.1329727 -4.621706     0.000738 0.0008         *  # Both tests signif.

$BC
[1] NA
```
