# Supplementary material for: A temporal beta‐diversity index to identify sites that have changed in exceptional ways in space–time surveys
Source: Ecol Evol. 2019 Feb 18;9(6):3500–14. doi: 10.1002/ece3.4984 (PMC6434560; doi:10.1002/ece3.4984)
Supplement: Supplementary file 4 [file ECE3-9-3500-s004.pdf]

Appendix S4

RESULTS OF CALCULATIONS WITH R FUNCTION TBI(), CHESAPEAKE BAY DATA

TBI tests of significance of the difference between years at each site and BCD,mat matrix containing the *B* and *C* statistics used to construct the B-C plot (Fig. 4); 25 brackish sites, years 2005 and 2008, fall survey data. Sites 4 and 8 had no species in common between 2005 (T1) and 2008 (T2). Significant adjusted p-values (Holm correction) are underscored in the results below.

1. Comparison based upon species abundance data, percentage difference D

```
( res.fauna.05.08.pcdiff = TBI(Y1, Y2, "%diff", pa.tr=FALSE, nperm=99999, BCD=TRUE,
test.BC=TRUE, test.t.perm=TRUE, clock=TRUE) )
# Computation time = 604.271000  sec

-----
$TBI
 [1] 0.6766467 0.5704698 0.9411765 1.0000000 0.6309524 0.7685950 1.0000000 1.0000000
 [9] 0.6960000 0.5777778 0.8632812 0.5081967 0.3229572 0.7083333 0.5164835 0.5843137
[17] 1.0000000 0.8983051 0.6385965 0.4244604 0.6256158 0.3846154 1.0000000 0.8020833
[25] 0.7611940

$p.TBI
 [1] 0.38334 0.61550 0.00627 0.00062 0.48268 0.19719 0.00062 0.00056 0.34265 0.59802
[11] 0.06230 0.74991 0.97718 0.31635 0.72990 0.58333 0.00064 0.03035 0.46790 0.89258
[21] 0.49572 0.93799 0.00058 0.14253 0.20731

$p.adj
 [1] 1.00000 1.00000 0.12540 0.01426 1.00000 1.00000 0.01426 0.01400 1.00000 1.00000
[11] 1.00000 1.00000 1.00000 1.00000 1.00000 1.00000 0.01426 0.57665 1.00000 1.00000
[21] 1.00000 1.00000 0.01400 1.00000 1.00000

$BCD.mat
        B/(2A+B+C) C/(2A+B+C) D=(B+C)/(2A+B+C) Change
Site.1  0.167664671 0.50898204        0.6766467    +
Site.2  0.436241611 0.13422819        0.5704698    -
Site.3  0.029411765 0.91176471        0.9411765    +  * Mostly abundances-per-sp. gains
Site.4  0.250000000 0.75000000        1.0000000    +  * Mostly abundances-per-sp. gains
Site.5  0.053571429 0.57738095        0.6309524    +
Site.6  0.347107438 0.42148760        0.7685950    +
Site.7  0.950000000 0.05000000        1.0000000    -  * Mostly abundances-per-sp. losses
Site.8  0.400000000 0.60000000        1.0000000    +  * 40% Ab.-per-sp. losses, 60% gains
Site.9  0.576000000 0.12000000        0.6960000    -
Site.10 0.100000000 0.47777778        0.5777778    +
Site.11 0.224609375 0.63867188        0.8632812    +
Site.12 0.483606557 0.02459016        0.5081967    -
Site.13 0.190661479 0.13229572        0.3229572    -
Site.14 0.229166667 0.47916667        0.7083333    +
Site.15 0.340659341 0.17582418        0.5164835    -
Site.16 0.482352941 0.10196078        0.5843137    -
Site.17 0.000000000 1.00000000        1.0000000    +  * Only abundances-per-sp. gains
Site.18 0.135593220 0.76271186        0.8983051    +
Site.19 0.057894737 0.58070175        0.6385965    +
Site.20 0.316546763 0.10791367        0.4244604    -
Site.21 0.571428571 0.05418719        0.6256158    -
Site.22 0.161538462 0.22307692        0.3846154    +
Site.23 0.098039216 0.90196078        1.0000000    +  * Mostly abundances-per-sp. gains
Site.24 0.005208333 0.79687500        0.8020833    +
Site.25 0.008955224 0.75223881        0.7611940    +
```

```
$BCD.summary
 mean(B/den) mean(C/den)   mean(D)   B/(B+C)   C/(B+C) Change
   0.2646503   0.4513519 0.7160022 0.3696222 0.6303778    +

$t.test_B.C, nperm=99999
               mean(C-B)      Stat    p.param  p.perm   p<=0.05
Paired t.test   0.1867016  1.826046 0.08031088 0.07998

$BC
[1] NA
-----
```

Note – The site names, Site.1 to Site.25, found in the function output file, correspond to the following site names on the Chesapeake Bay map:

```
site.names
 [1] "S1"   "S15"  "S201" "S202" "S203" "S204" "S22"  "S23"  "S24"  "S26"
[11] "S29"  "S40"  "S43"  "S44"  "S47"  "S51"  "S52"  "S6"   "S62"  "S64"
[21] "S66"  "S68"  "S71"  "S74"  "S77"
```

---

2. Comparison based upon species occurrence (i.e. presence-absence) data, Sørensen D

```
( res.fauna.05.08.sor = TBI(Y1, Y2, "sorensen", pa.tr=FALSE, nperm=9999, BCD=TRUE,
test.BC=TRUE, test.t.perm=TRUE, clock=TRUE) )
# Computation time = 45.459000  sec

-----
$TBI
 [1] 0.4838710 0.4166667 0.6666667 1.0000000 0.2727273 0.1578947 1.0000000 1.0000000
 [9] 0.3846154 0.1818182 0.3600000 0.3333333 0.2500000 0.4000000 0.2500000 0.2857143
[17] 1.0000000 0.5238095 0.2592593 0.2592593 0.3333333 0.1538462 1.0000000 0.1304348
[25] 0.2592593

$p.TBI
 [1] 0.1607 0.2984 0.0217 0.0010 0.7423 0.9574 0.0005 0.0004 0.3902 0.9261 0.4807
[12] 0.5910 0.8108 0.3526 0.8042 0.7025 0.0009 0.1202 0.7714 0.7748 0.5940 0.9593
[23] 0.0006 0.9768 0.7727

$p.adj
 [1] 1.0000 1.0000 0.4340 0.0210 1.0000 1.0000 0.0120 0.0100 1.0000 1.0000 1.0000
[12] 1.0000 1.0000 1.0000 1.0000 1.0000 0.0198 1.0000 1.0000 1.0000 1.0000 1.0000
[23] 0.0138 1.0000 1.0000

$BCD.mat
        B/(2A+B+C) C/(2A+B+C) D=(B+C)/(2A+B+C) Change
Site.1  0.06451613 0.41935484        0.4838710    +
Site.2  0.25000000 0.16666667        0.4166667    -
Site.3  0.16666667 0.50000000        0.6666667    +
Site.4  0.50000000 0.50000000        1.0000000    0  * Equal numbers of losses and gains
Site.5  0.09090909 0.18181818        0.2727273    +
Site.6  0.15789474 0.00000000        0.1578947    -
Site.7  0.87500000 0.12500000        1.0000000    -  * Mostly species losses
Site.8  0.50000000 0.50000000        1.0000000    0  * Equal numbers of losses and gains
Site.9  0.15384615 0.23076923        0.3846154    +
Site.10 0.09090909 0.09090909        0.1818182    0
Site.11 0.16000000 0.20000000        0.3600000    +
Site.12 0.22222222 0.11111111        0.3333333    -
Site.13 0.08333333 0.16666667        0.2500000    +
Site.14 0.20000000 0.20000000        0.4000000    0
Site.15 0.12500000 0.12500000        0.2500000    0
Site.16 0.14285714 0.14285714        0.2857143    0
Site.17 0.00000000 1.00000000        1.0000000    +  * Only species gains
Site.18 0.38095238 0.14285714        0.5238095    -
Site.19 0.11111111 0.14814815        0.2592593    +
Site.20 0.11111111 0.14814815        0.2592593    +
Site.21 0.28571429 0.04761905        0.3333333    -
Site.22 0.03846154 0.11538462        0.1538462    +
Site.23 0.36363636 0.63636364        1.0000000    +  * Mostly species gains
Site.24 0.04347826 0.08695652        0.1304348    +
Site.25 0.11111111 0.14814815        0.2592593    +

$BCD.summary
 mean(B/den) mean(C/den)   mean(D)  B/(B+C)  C/(B+C) Change
   0.2091492   0.2453511 0.4545004 0.460174 0.539826    +

$t.test_B.C
               mean(C-B)       Stat   p.param  p.perm   p<=0.05
Paired t.test   0.0362019  0.6218665 0.5398928  0.5560

$BC
[1] NA
```
